# Micro aerial vehicle with basic risk of operation

**DOI:** 10.1038/s41598-022-17014-4

**Published:** 2022-07-27

**Authors:** Alysson Nascimento de Lucena, Bruno Marques Ferreira da Silva, Luiz Marcos Garcia Gonçalves

**Affiliations:** 1 Universidade Federal do Rio Grande do Norte, Graduate Program in Electrical and Computer Engineering, Natal, 59078-970 Brazil; 2 Universidade Federal do Rio Grande do Norte, Graduate Program in Mechatronic Engineering, Natal, 59078-970 Brazil

**Keywords:** Aerospace engineering, Electrical and electronic engineering, Mechanical engineering

## Abstract

We draw current efforts towards proposing a wing-type micro UAV with characteristics of being a basic operation risk self handled (Micro-Brosh) platform. Its micro-sized wingspan and weight, which are less than 0.30 m and 0.150 kg, respectively, guarantee the low risk to the operator and installations in case of crashing. It can be launched manually without using an appropriate runway, besides using a soft grass field for landing is recommended. Its associated costs for construction and maintenance are very low (below US$ 500) if compared to traditional aircraft. The main contribution here is the architectural design, besides we provide detailed documentation including techniques for determining lift, thrust, drag, minimum flight velocity, maximum time of flight and distance (autonomy), and other issues that we have solved related to the aircraft development. We validate our prototype with experiments including several tests done with the UAV with results that have proven its flight ability. To the end, we understand that this work provides a nice starting document to researchers that intend to develop a UAV or enter this field.

## Introduction

Unmanned aerial vehicles (UAV), also called Unmanned Aerial Systems (UAS), have applicability in several tasks such as monitoring and pest control in agriculture ^1–4^, images acquisition for geological survey, mining, and photogrammetry ^5–8^, disasters monitoring, searching, and rescuing ^9–11^, monitoring of atmosphere ^12,13^, surveying terrestrial and aquatic wildlife ^14,15^, inspection of lines and structures in smart cities ^16,17^, environmental monitoring^18,19^, and maritime surveillance^20^, between several others. In relation to the mechanical design and construction of UAVs, they can be roughly divided into two classes, which are (1) UAVs with fixed wing and (2) the ones with rotating wings. Notice that each class has inherent properties with a better applicability that depends on the type of application task ^21^. Single or multi-rotor UAVs are the most common drones encountered in the market due to their easy of use and maneuverability capability. It provides hovering literally stopping in the air, and vertical takeoff and landing. Nevertheless, its energy consumption is generally high, thus it has limited autonomy of the flight (distance and flight duration). This is not the main problem in the UAVs with fixed wing, mainly the ones with combustion engines that can cover larger distances and performing flights with longer duration. Even electrical ones can be more efficient that multi-rotor ones in these requirements of travelled distance and flight time. This is mainly because the energy matrix necessary for the lift is smaller. The use of a wing with a certain minimal velocity provides the necessary lift with less power consumption than the multi-rotor. At lastly, we can add the hybrid class (3) that appears joining both previous (1) and (2) classes in a single UAV system able to perform both vertical and horizontal flights with the same platform ^22^. In this case, a single or multi-rotor propeller, in general electrically powered, provides the lift necessary in vertical takeoff and landing and also on hovering in the air. Notice that hovering could be useful in missions that deserve a stopping in the air, as for example for better observing some desired feature. It is also able to perform horizontal flight by way of using electrical or combustion propellers that act on the wing surface to provide the lift that supports the aircraft in the air and at the same time pushes it forward. Notice that this UAV type should have a robust control system, especially for providing a smooth transition between vertical and horizontal flight configurations. In this class, the aircraft vary from the ones that are similar to a traditional airplane to flying wings. The ones with more autonomy of flight, in general, use combustion motors besides completely electrical versions exist. Hybrid UAV models are still a matter of research and development ^23^, besides some of them are available at production and operation ^24–29^.

Our study in this work is about one specific type, classified as micro-UAV (or simply MAV) that falls into the first class (1), with fixed wing. We have chosen this type mainly considering its simpler development cycle and the possibility of diminishing damages caused by accidents, which is easier to be controlled in the case of these very very small UAV. Actually, our work is motivated by a research project that we submitted and that has been granted by a public call of the Brazilian sponsoring agency FINEP ^30^. The project core is the development of a micro-UAV prototype that fulfills the basic specifications of the call, which is basically to deploy a UAV with 0.200kg that can fly for about one hour and reach a distance of 10km. Diminishing the probability of damages caused by accidents is also desired in our project and this is one of the main concerns nowadays, in regarding of the increasing rate and variety of uses of UAVs. Several researchers have worked on predicting such dangerous situations for humans ^31^, also on the development of anti-collision systems^32–34^, using systems with dumping on fall for diminishing impact^35,36^, and even using airbag systems^37^. In parallel to these developments, governs worldwide, in general guided or supported by the scientific community, have proposed and sanctioned their regulatory laws following the evolution on the development and use of UAV ^38,39^. For example, Brazilian national agency for civil aviation (ANAC) has published since 2017 the General Requirements for Unmanned Aircraft for Civil Use (shortened as RBAC-E-94). These rules divide the use of UAVs in two classes: (1) recreational and (2) commercial or corporate. Also, there are different requirements according to other 3 classes that are defined regarding the maximum weight allowed on takeoff (MTW). Our proposal has a weight below 0.150 kg, which fits in class 3 (up to 0.250 kg). In this class, most of the rules requirements following RBAC-E-94 ^40^ are disregarded but one, which states that the age of the operator should be above 18 years old. These adopted rules resembles the requirements of the MAV competition ^41–43^. Hence, our main proposal is to develop a fixed wing micro-UAV that weights up to 0.200 kg and can flight one hour and 10 km providing at the same time very low risk to installations and humans. We shortened all of these characteristics as Micro-B.R.O.S.H UAV (or Micro Basic Risk Operation Self Handled UAV), being this theme one of the hot topics in the Unmanned Aircraft Vehicles field of research ^31,44^.

Brandt et al. ^44^ describe a way for selecting a low-risk UAV (Unmanned Aerial Vehicle) configuration including a methodology for determining the size and optimization of the shape. These researchers work with both combustion propelled and electric propelled UAV. Electric-powered aircraft is described to the extent that it differs from that for fueled aircraft. With the design of a small UAV, they verify the methodology for some required performance and payload. Our approach has been designed somewhat following this model, with several intermediary prototypes built in order to reach the best one. Hence, we use an experimental design with a prototyping paradigm, making several, different models of UAV during our studies, using cutting-edge technology. We also have done theoretical studies based on simulation for determining most parts of the architectural design, in order to solve instability caused by wind gusts during flight and other smaller problems that appeared. To date, we have a working prototype that has shown to be effective in the several flights that we perform during tests. All prototypes have been tested in extreme wind conditions that happen in the northeast of Brazil and the chosen one has demonstrated stability in the presence of gusts, thus satisfying our requirements. Its first drafts have been submitted to a patent^45^ and firstly published as a conference paper ^46^. The previous work has been further enhanced in all of its aspects, with new developments and experimental results that we introduce in the current manuscript. Also, in order to make our work reproducible, here we describe our methodological approach in a more detailed way.

Thus, our general goal in this work is to move a step forward towards the architectural design of a UAV with very reduced size and weight that can diminish damages to installations and humans. We can enumerate the several several contributions of the work as:

1. The main scientific contribution is the architectural design of the MAV, with detailed documentation including techniques for determining lift, thrust, drag, minimum flight velocity, maximum time of flight and distance (autonomy), and other issues that we have to solve related to the aircraft development.
2. As a technological product, we propose and have built a wing-type micro UAV with characteristics of being a basic operation risk self handled (Micro-Brosh) platform.
3. Our prototype has been validated with practical experiments including several tests done with the UAV with results that have proven its flight capability.
4. As a final (technical) contribution, we understand that this work provides a nice starting document to researchers that intend to develop a UAV or enter this field.

Our current solution has a weight ranging from 0.080 kg to 0.200 kg, which depends on the amount of embarked devices and payload. Based on experimental research where we have made several prototypes, we choose a wing type UAV with a wingspan between 0.20 m and 0.30 cm. The single propeller that pushes the UAV is localized in the center of gravity and is protected by most part of the aircraft, which reduces probability of damages in accidents that may eventually occur, mainly, during takeoff or during the flight. However, a configuration where the propeller is installed at the tail is also possible. This is a very desired characteristic that makes this model a good choice for applications in populated areas. In the following we describe the experimental research and studies that have been done, coming up with the design and construction of the final prototype.

## Related works

Multi-rotors UAV, mostly known as *drones*, are the most used small UAV nowadays because they have a simpler control and an easier to handle process than fixed wing UAV. Vertical takeoff and landing and hovering capabilities are their main advantages. However, it is well known that they lack a long range flight capability ^47^ being the fixed wing type a better choice when this is a requirement. Nevertheless, there is a difficulty with fixed wing UAV when going to even smaller scales^48^, which is our main requirement here. A substantial part of the lift is compromised when the wing area becomes very small due to the aerodynamics of the structure. Known wing profiles with small areas on very small aircraft systems lead to Reynolds numbers as low as 10,000–200,000, what causes a bigger influence on the flight stability. This problem happens mainly in the presence of certain weather conditions such as subtle variation in air temperature, humidity, and pressure. Hence, these smaller scale airplanes require more studies and developmental efforts and this has being one of the the hot topics studied nowadays, besides not been as recent ^49^. Besides different classifications for UAV appears in the literature ^47^, for the sake of simplicity in this work, we adopt our own classification considering the wingspan and weight. According to the classification adopted, an aircraft can be regarded as being of large (or big) size (1), small size (2), very small (or miniature) size (3) and micro UAV or MAV (4).

### Large and small size UAVs

**Figure 1:**
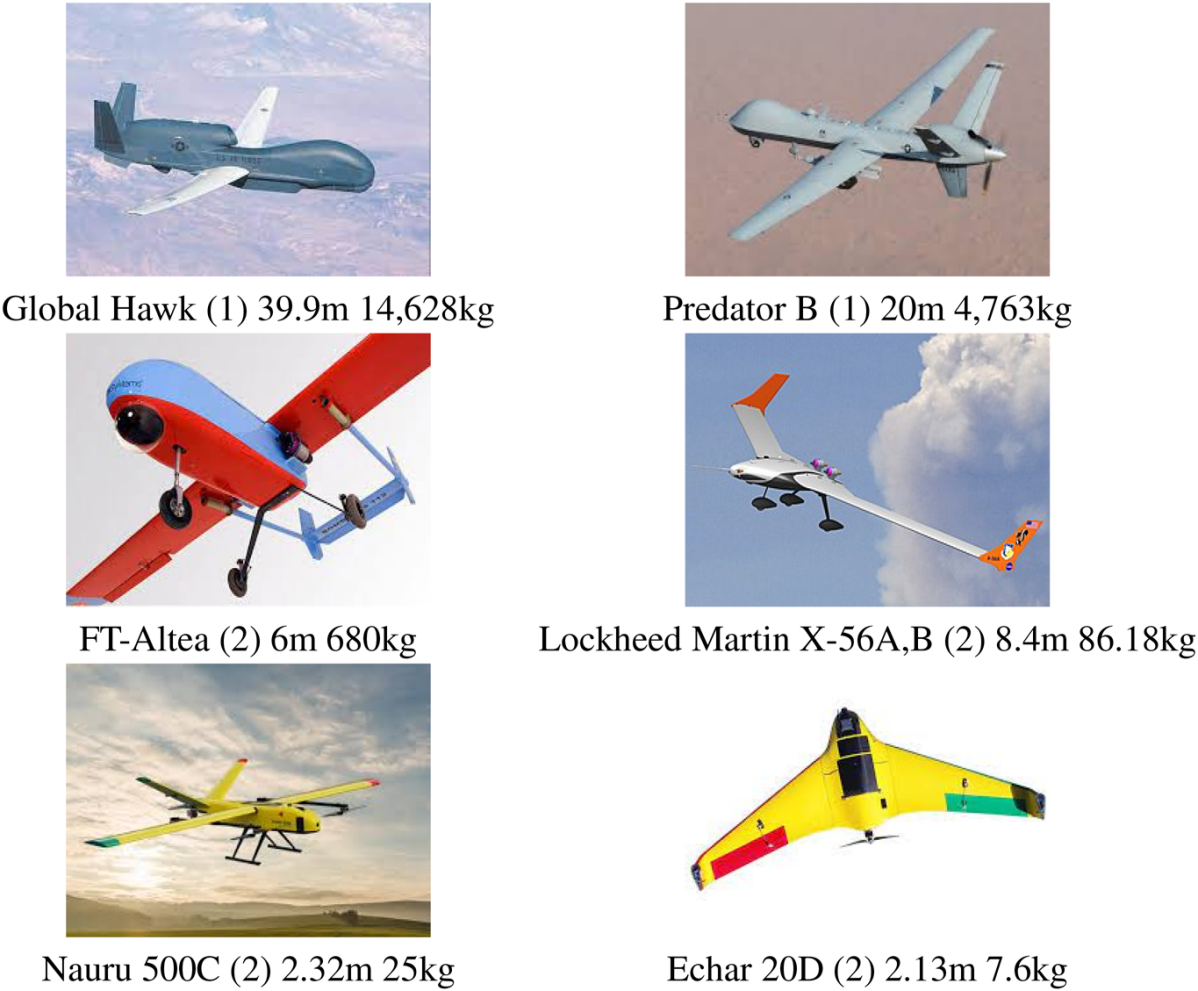
Main fixed wing UAV systems in classes (1) big and (2) small.

Figure 1 shows some selected UAVs on large (1) and small (2) sizes. One of the most famous large size UAV is the Global Hawk, which has a wingspan of 39.9 m and can takeoff with a maximum weight of 14.6 tons, being manufactured by Northrop Grumman. A second UAV also of similar application and characteristics is the Predator-B that has 20 m of wingspan and is able to takeoff with 4.7 tons, being manufactured by General Atomics. Between the small size UAVs (2), we can highlight the FT-Altea weighting 80kg and with a wingspan of 6m, manufactured by Flightech Systems Europe^50^, the Lockheed martin X-56 A and B, with 8.4 m of wingspan and approximately 86 kg, and the Nauru 500C that has 2.32 m of wingspan and allows a maximum takeoff weight of 25 kg, produced by XMobots^51^. Aircrafts as the ones listed above generate several concerns being regulated by aeronautical organisms in most countries and requiring large structures and logistics for their operations, which limits their applications. In general, they need adequate runways for taking off and landing and have high acquisition and maintenance costs. Besides, it is relatively easy to detect and track them, and this could be an advantage or a problem depending on the application. Nevertheless, most concerns of the regulatory authorities are related to the safety of both people and installations on the operation area, mainly when they fly over populated areas. Depending on the application they can be as dangerous or more dangerous than a manned aircraft.

### Very small size UAVs

**Figure 2:**
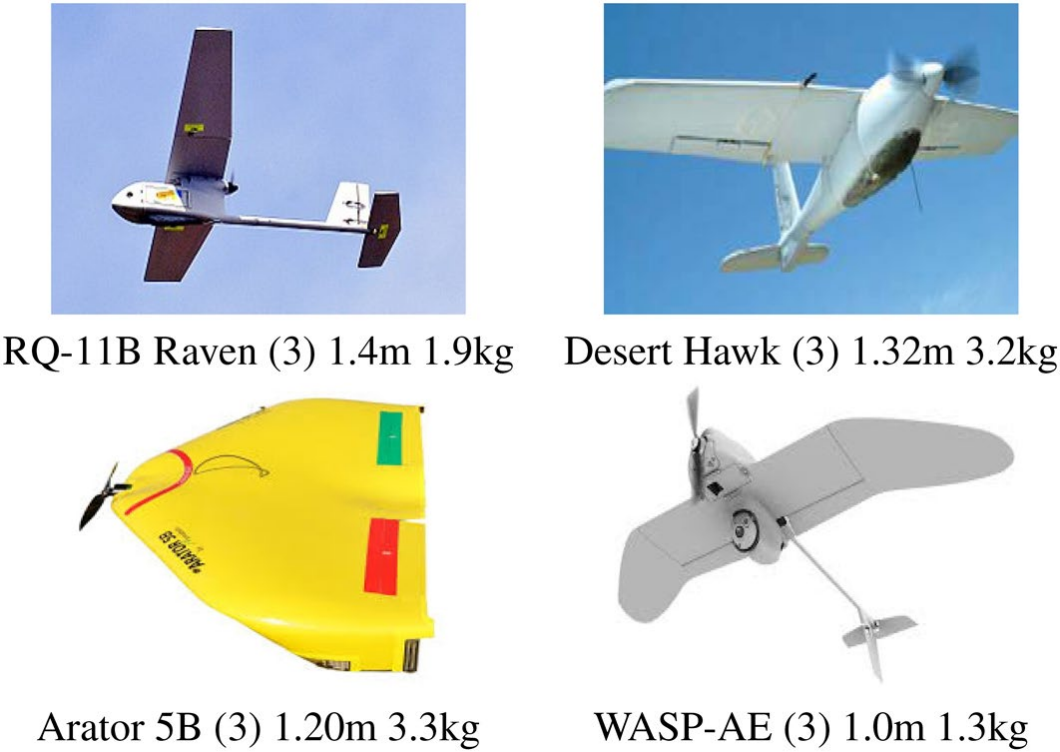
Main fixed wing UAV systems considered as very small (3) in the current work.

On diminishing the size, we go to even smaller aircraft considered as very small here or (3) in Fig. 2, with wingspan between 1.0 m and 2.0 m and weight between 1 kg and 5 kg. Their main use is regarded to entertainment and military (surveillance) applications. They have low payload and a reduced flight time, besides some times they respond to the needs of some specific project, depending on the task. In general, these UAVs have electric power-trains and takeoff is executed by way of throwing them in the air by hand or by using some elastic impulse. There are several projects with these characteristics, developed for the most diverse serious applications ^52–55^. In fact, the pursuing on miniaturization can be partially regarded to applications in the US army, which has used very small airplanes since 1999. The first one is known to be the RQ-11 Raven ^54^ developed by AeroVironment. With 1.4 m of wingspan and weight of 1.9kg, it is one of the most deployed UAVs of the world, mainly used in the war, however also including other applications. It has some 90 min of flight time with batteries and requires a single operator for its assembly and launching. The Desert Hawk is another very small sized UAV, with 1.32 m of wingspan and weighing 3.17 kg, which has been developed by Lockheed Martin ^55^. It has roughly one hour of flight autonomy and it can be launched by way of using a bungee cord besides by hand. We observe that these very small sized fixed wing UAVs with aircraft model varying from traditional airplanes to flying wings are already well developed ^52,54–57^. Their use, in general coupled with RTK and imaging systems, represents an already settled technologically and innovative practice and they are commercially available nowadays.

### From miniature to micro UAV systems

**Figure 3:**
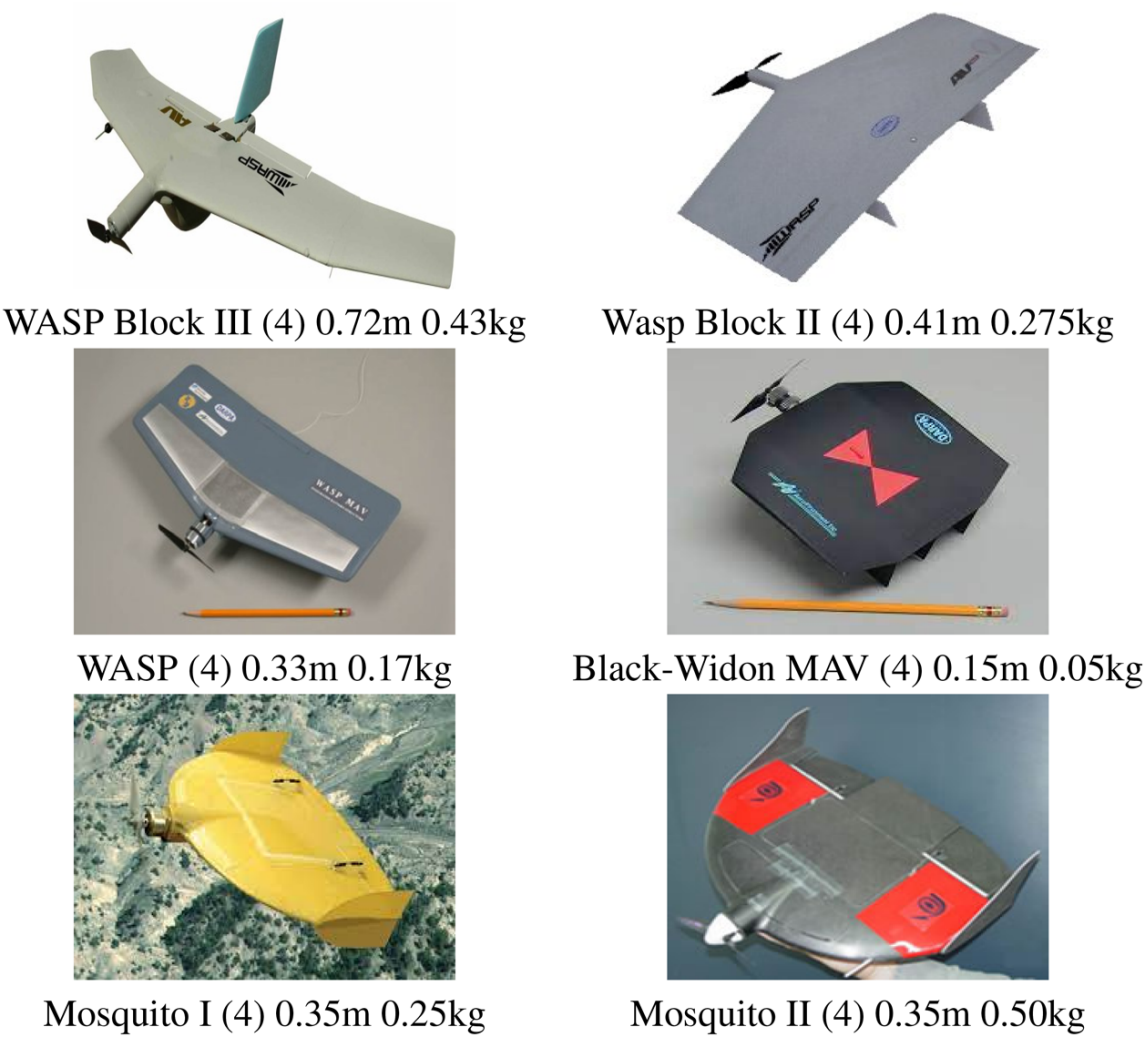
Main fixed wing that we consider in class (4) in the current work, from miniature to micro UAV (MAV) systems.

The basic difference of the above works and the present proposal is that the aircraft here proposed should be even much smaller, for attending our project requisites and not offering danger to its operator nor to humans or installations that may exist in the area of operation. We performed an extensive search in the literature in which we could find several approaches dealing with aircraft varying from miniature to micro UAV (or MAV), as seen in Fig. 3. We observe that none of them are strictly similar to ours considering its operation mode and size. Besides, we notice a fast growing in the last decade, on the number of researches dealing with MAV, treating separately fixed-wing and VTOL UAVs and also the hybrid ones ^58^. We observe that most of them have limitations in flexibility, time of flight, and payload capability, being not adequate for several tasks, mainly tasks with long duration.

**Figure 4:**
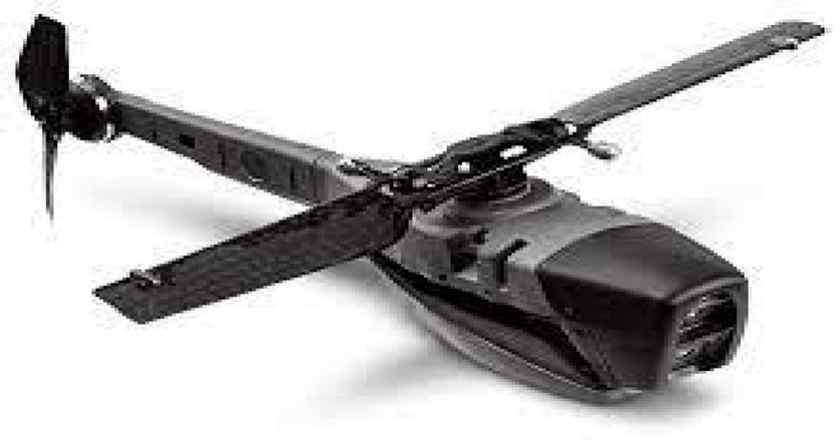
The Black Hornet micro UAV, it partially fits the needs of our project, but flight distance. Black Hornet measure is 0.168 cm, overall, and its weight is 0.033 kg.

Our results on searching smaller UAV projects include a survey paper in which the authors produce a very good summary from 132 available prototypes, which is still valid nowadays besides not being a very recent work ^47^. It refers to the Black-Widon MAV, with only 0.015 m as seen in Fig. 3, as one of the earliest fixed-wing micro UAVs, which has been developed in 1999 by AeroVironment ^47^. The first prototype of Black Widon weights 0.056 kg, and has performed a flight of 22 min at a cruise speed of 40 km/h transmitting a black and white video ^59^. The last version reported has weight of 0.080 kg and is able to perform flights up to 30 min of endurance at a maximum altitude of 235 m, however with a maximum range of 1.8 km, which fits out of our desired range ^59^. Since then, the Black-Widon project has been renamed to WASP with some optimization on the wing structure and diminishing the battery consumption ^47^. As said, the smaller version has been replaced by extended ones with sizes of at least 0.33 m, named as Wasp, Wasp Block II and Wasp Block III in Fig. 3. Notice that this is bigger than ours. Besides the Wasp series has other strong similarities to our proposal, we consider that Mosquito is the most close to ours ^60^, mainly because of its larger operation range. This UAV has been developed by Israel Aerospace Industries (IAI), in two versions, 1.0 and 1.5, currently. The two versions have a wingspan of 0.33 m. Their weights are 0.25 kg for the 1.0 and 0.5 kg for the 1.5 version, so equals or bigger to ours. This MAV can fly with a maximum speed of 46 km/h at an altitude of 200 m. Nonetheless, we find out that it can fly in a range of 3 km around the base station, besides being able to perform beyond visual line of sight (BVLOS) flight. It is robust in worst wind conditions facing wind gusts up to 25 knots and its flight duration is about 40 min. Mosquito delivers live streaming with multiple cameras, and has GNSS besides other capabilities. We observe at this point that the above MAVs, but the Black Widon, have wing size above 0.30 m, which is one of our main requirements (actually, ours can be 0.25 m). Nevertheless, we also found other platforms in the rotating wing class, as the Micro-Mosquito and the Black Hornet. Micro-Mosquito is a double helix helicopter toy that is commercially available and has no UAV characteristics, being remotely controlled. The Black-Hornet, seen in Fig. 4, is a micro helicopter that has been developed by Prox Dynamics with full UAV characteristics. It has only 0.033 kg and can fly about 25 min at a speed up to 10 m/s, with BVLOS capability and a range up to 2 km. Considered by the UAV community as a fully autonomous MAV, it can provide video and image transmission in real-time.

### Strictly related works

As introduced in our previous conference work ^61^, the basis for our UAV system is a fixed wing shape, weighting up to 0.200 kg, with a wing size of 0.25 m, and autonomy of one hour of flight in a 10 km range. Hence, overall, we found five works with close characteristics, that mostly resemble ours ^59,60,62–64^. The first work ^62^ is the most recent project ^62^ having a wingspan 0.45 m a and weight of 104 g. It can fly with a cruising speed of 7 m/s and with autonomy of about 2.8 h of flight duration. The second prototype that we found also resembling ours, however being out of our scope, has a length of 0.38 m and wingspan of 0.91 m, with a 1.15 kg of weight ^63^. A not last work close to our intents is an aircraft project named *Bidule* that has started in 1998. This work has since then been revisited only in 2006, nevertheless. We found a wingspan of 0.41 m and no weight is reported in their later manuscript ^64^. The Black Widon ^59^, described above, has been discontinued, probably due to its limited applicability. Its producer company has created several other bigger sizes UAV that are currently commercially available. Information about the last work (Mosquito) is available in a web page in which we could not find articles nor technical documentation describing its detailed technical issues, which is essential for reproducibility ^60^.

Hence, we definitely select the Black Widon and Mosquito UAVs as the works that we believe are with nearest characteristics to ours ^59,60^. This includes part of the architectural design as the shape in the form of a flying wing, however, as said above, our UAV is even smaller than the Mosquito with a wingspan of 0.25 m, what makes it possible to have a greater operation range than the Black Widon. Our first prototype could fly at cruising speed of 12.5 m/s, with some 20 min of flight duration, or 15 km. Actually, this topic is one of the priorities on our current research and we have improved this autonomy in the current version, going to some 45 min (estimated). After all, the main difference that we can point out from our proposal and both similar UAVs is the propeller positioning, which is on its center of gravity (or in the back) that we believe makes it safer than Mosquito and Black Widon. Notice that this offers less danger to installations nor to its operator or other person, in case of accident.

Lastly, we remark a feature that is a default consequence of micro sized UAV, which is the low radar cross section (RCS) also called radar signature. This is a feature thought after in surveillance applications, which is mostly defined based on the aircraft shape ^65^. For example, this is a main characteristic for reconnaissance and bomber aircraft projects, as the long-range SR-71 ^66^, F-117 Nighthawk bomber, B-2 Spirit bomber, F22 aerial superiority fighter, and the F35 Multi-Purpose Fighter, besides being big sizes UAV. Our proposal has low RCS (radar response) and is almost visually imperceptible, mainly at distances greater than 400 m. Notice that this is a natural characteristic due to its micro dimensions, which also makes easy its camouflage after use.

## Methodology for architectural design and construction

There are several restrictions to consider in the UAV architectural design. The first ones are imposed by the rules of civil aviation organisms as the ANAC RBAC-E Nr 94 ^40^, in Brazil, which includes the several aspects in each category as shown in Table 1. Our project call requires a UAV that falls into the third class (3), however imposing a maximum weight of 0.200 kg. With this we avoid certification for most of the restrictions, but one, as it can be seen in the table, which is the operator age above 18 years. Hence, restrictions as low risk to installations and humans, besides the specifications in size, weight, and flight autonomy are the main issues to be observed in the conceptual design. Other secondary issues to be observed are easy of assembly/construction and low-cost.

**Table 1:**
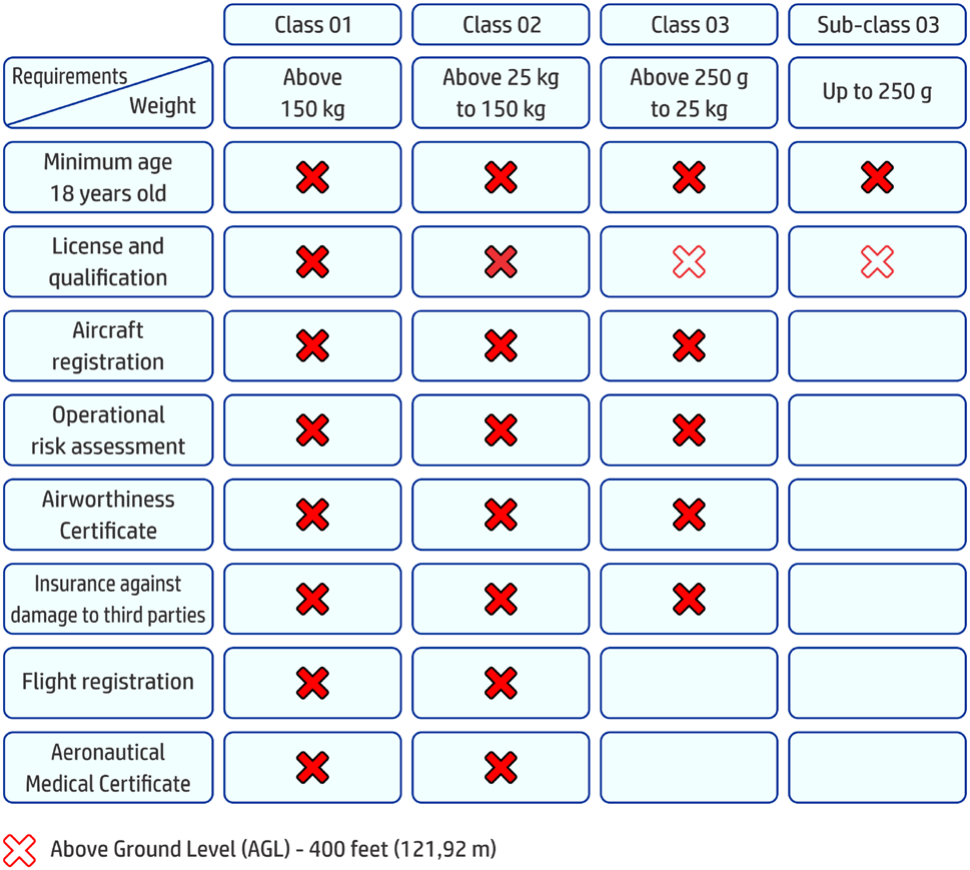
Classes and requirements of ANAC accounted for the MicroBrosh UAV project.

### Choosing the right prototype model

After analyzing the several works seen above, we can see that fixed wing platform is a better choice than rotatory one due its larger autonomy, both in distance and time of flight. Nonetheless, we still have to choose the right architectural design. Basically, we have to select between the several aircraft shapes varying from airplane like to flying wing ones, in outdoor conditions. For that, we have done a pure experimental (practical) research, where we have constructed a series of prototypes following the most common designs available in the literature and performed flights with them. In total, we built more than 15 prototypes, which resulted in 9 basic models, with some of them seen next. We notice that we destroyed some of them during our practical flights, due to several reasons, as the wind gusts (main one). All of these flights have been done first indoor and then outdoor, in severe wind conditions as will be depicted further in “Experimental setup” section. In the following we describe some of these models selected between the 9 prototypes, which performed best in the tests that will also be described later. We constructed all prototypes using extruded polystyrene.

**Figure 5:**
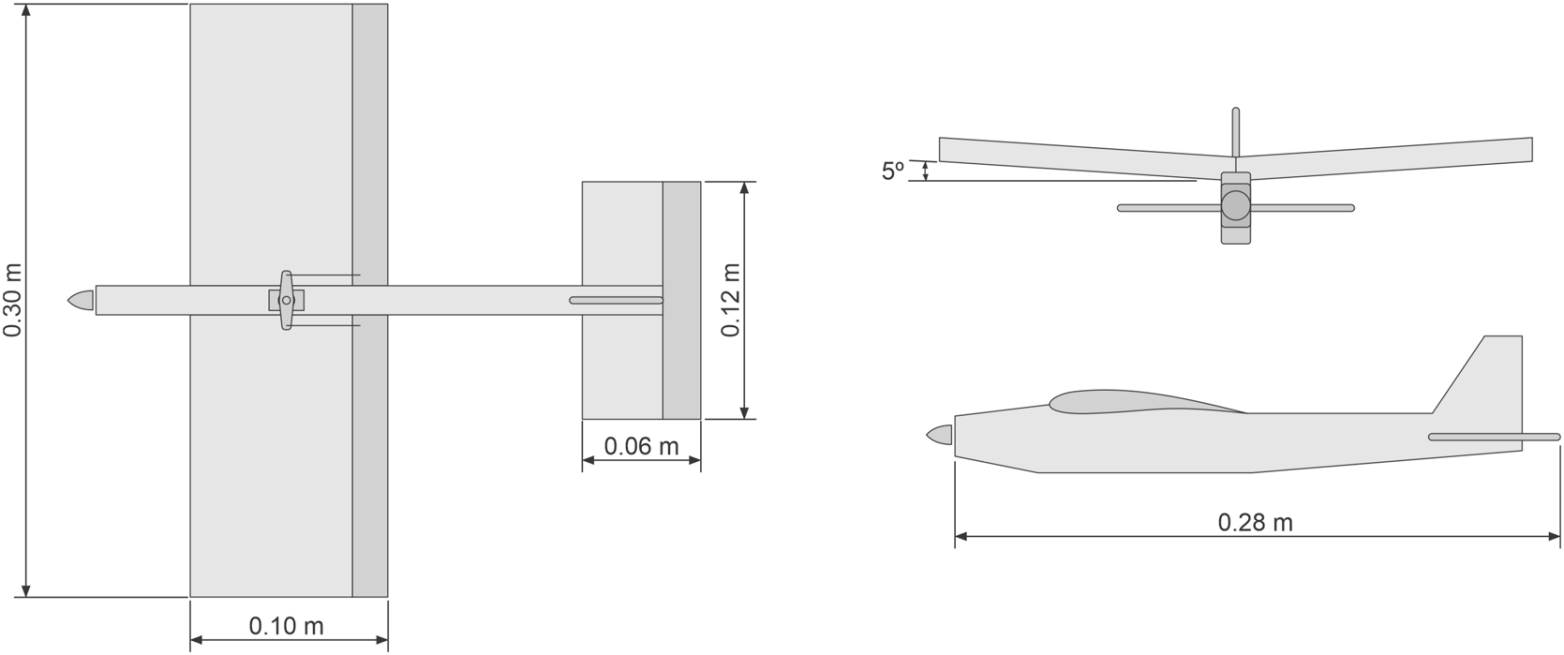
Prototype 4, made with extruded polystyrene.

Figure 5 shows the basic design of prototype 4 that is based on the Eppler 423 profile. It has massive wing with wingspan of 0.30 m and fuselage with length of 0.28 m, with a 0.10 m rope. Its total weight is 0.100kg, including a single micro brushless motor (5 g and 2000 kv) with a $4\times 2.5$ GWS propeller and 650 mA current HXT500 5 g micro servos, all powered by a 7.4 v lithium battery.

**Figure 6:**
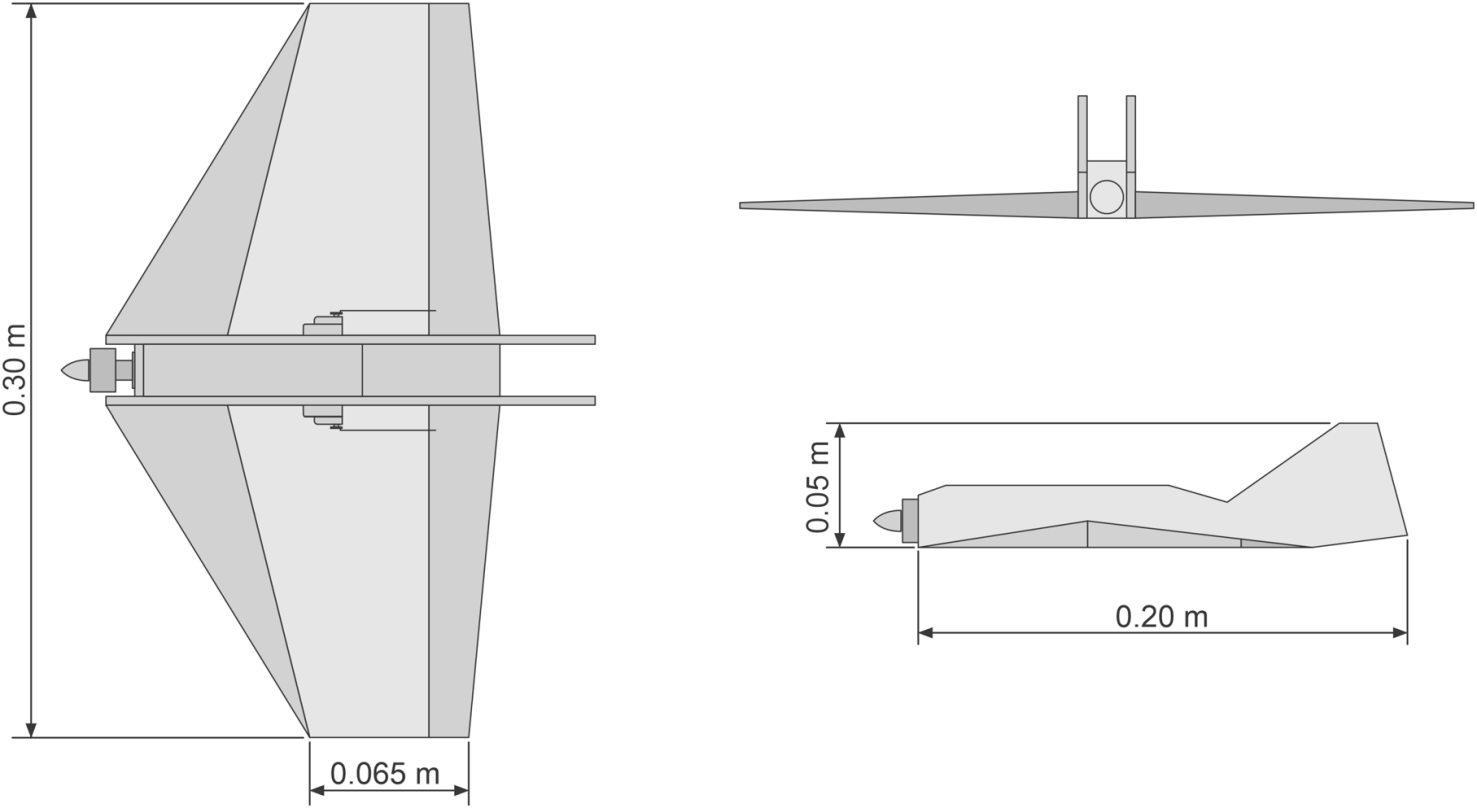
Prototype 5 is our first Delta shaped model tested, also made with extruded polystyrene.

Prototype 5 (Fig. 6) is the first delta shaped wing model tested, with 0.30 m wingspan, 0.11 m medium rope, and cave-like fuselage with length of 0.20 m. Its total weight is 0.110 kg, including a brushless micro motor (10 g and 2000 kv) for pushing an APC $7\times 4$ propeller, and HXT500 5 g micro servos, which are all powered by a lithium battery with 7.4v voltage and 650 mA current. We use an RC Corona RP 4S1 72MHz 4 channels receptor, and transmitter JR XP9303H 9 channels PCM standard for communication.

**Figure 7:**
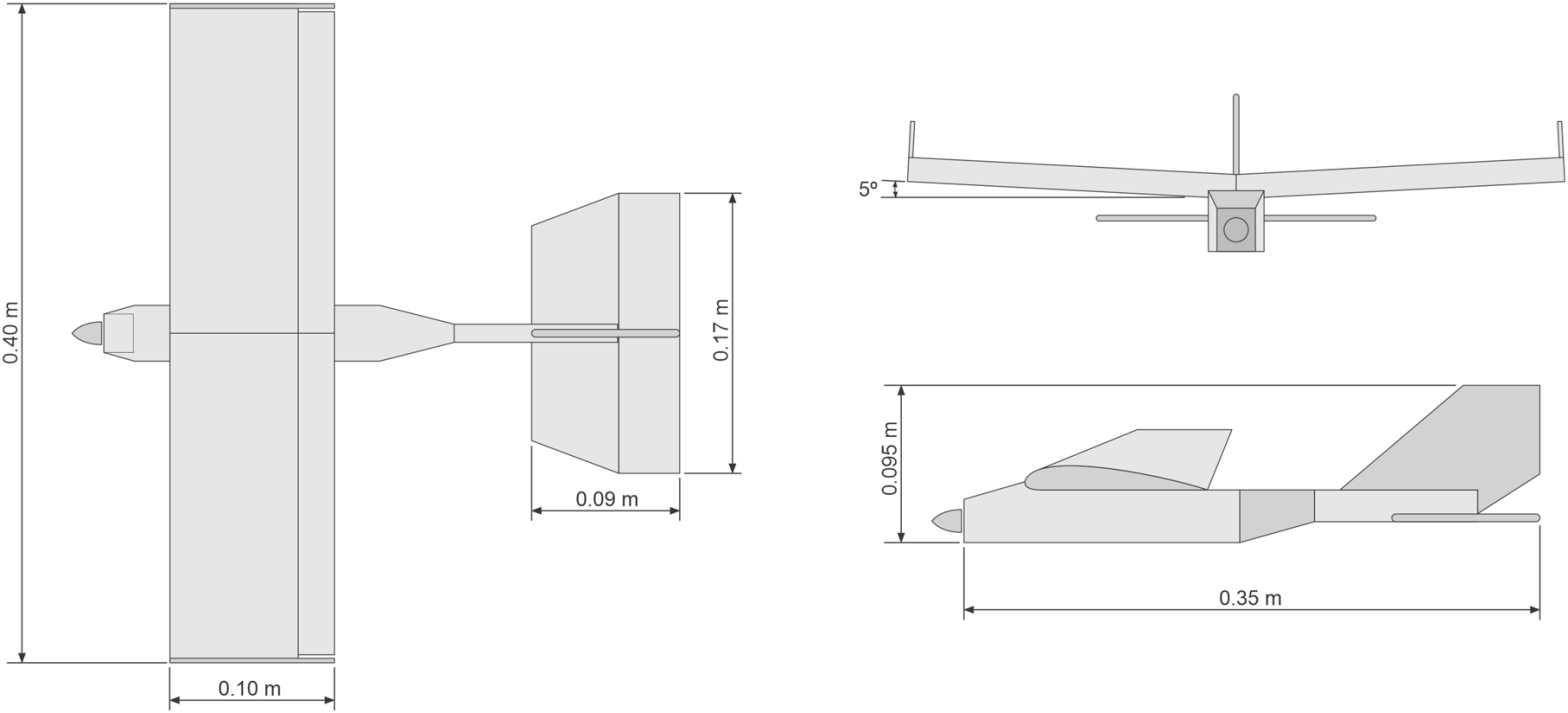
Prototype 6, in a top wing traditional model.

Next, we built prototype 6 following a default aircraft model with a top wing in a ribs/string pattern, based on the Bruxel 33 profile (Fig. 7). It has a 0.40 m wingspan and a 0.10 m rope, with a 0.35 m long cave-type fuselage, with 0.130 g total weight. A 10 g lithium battery with voltage of 7.4 v and current of 650 mA provides power for a 2000 kv micro brushless motor with an APC $7\times 4$ propeller and micro servos HXT500 5 g. For communication we use a 4-channel Corona RP 4S1 72 MHz receiver and JR XP9303H 9-channel standard PCM transmitter.

**Figure 8:**
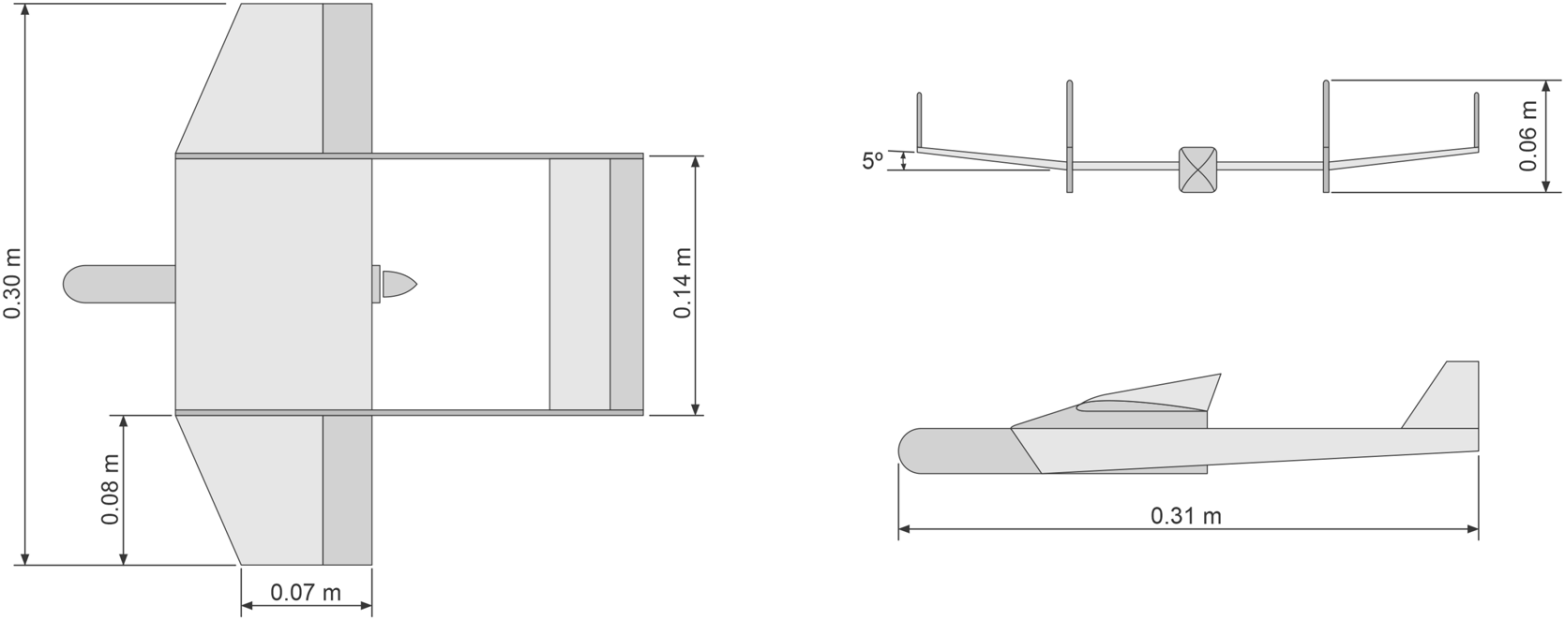
Prototype 8 in a top wing configuration aircraft model.

Prototype 8 (Fig. 8) also follows a top wing model, however with 0.30 m wingspan and 0.10 m average rope, and with a cave-type fuselage, in total a weight of 0.100kg. It has a 10g and 2000 kv micro brushless motor for the APC $7\times 4$ propeller and micro servos HXT500 5 g, all powered by a lithium battery with voltage of 7.4 v and current of 650 mA. We use a 4-channel Corona RP 4S1 72 MHz receiver and a JR XP9303H 9-channel standard PCM transmitter.

**Figure 9:**
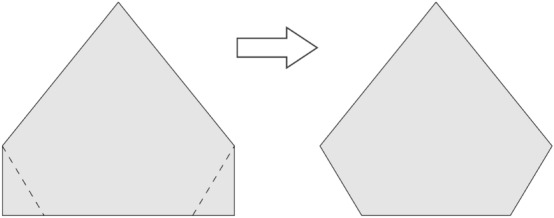
Conceived geometry of the final prototype 9 (in a $\Delta$ shape with a cut in each panel).

Prototype 9 (Fig. 9) is the last one that was designed, built, and tested (and chosen). Its shape, angles and dimensions were selected based on experiences with prototype 5, which has been modified. Among these major changes, the central chord was increased from 0.230 to 0.276 m with the aim of moving control surfaces away from the center of gravity, increasing the efficiency of controls and decreasing longitudinal instability during flight. This allows to increase efficiency in horizontal flight, improving performance.

The slope on the control surfaces causes them to act as a command mixing aileron with elevator. This was entered to compensate for the loss of lift generated during the rolling motion. This compensation can normally be applied by the autopilot, however, our solution alleviates the need for corrections, providing more stability to the aircraft in flight.

The choice of 35$^{\circ }$ of inclination of the control surface in relation to the trailing edge underwent a practical analysis of radio controlled flight, where the inclinations of 45$^{\circ }$, 35$^{\circ }$ and 25$^{\circ }$ were tested. Inclination of 35$^{\circ }$ has shown to be the one that presented the best correction, not requiring so much compensation by the pilot during the roll movement. The 25$^{\circ }$ option showed little improvement and the 45$^{\circ }$ option showed excessive interference. This inclination has a direct influence on the leading edge angle, since the lateral chord of the wing, in this case equal to zero, coincides with the inclined line of the control surfaces.

Hence, with a delta wing shapee derived from prototype 5, it is made with extruded polystyrene with 0.25 m wingspan, and cave-type fuselage, weighting 0.74 g in total. It has a 10 g, 2000 kv micro brushless motor, for pushing a $4.5\times 3$ APC propeller, and uses HXT500 5 g micro servos, all powered with lithium battery of 7.4 v voltage and 250 mA current. A 4-channel Corona RP 4S1 72 MHz receiver, JR XP9303H 9-channel PCM standard transmitter completes this model. We currently added the automatic pilot to it, as it will be seen next, and also in “Experimental setup” section. This model has the best stability between all of them, and has been chosen, so we start describing it in more detail in the following. The powertrain configuration (pusher) was based on prototype 8.

### Geometrical conceptualization

**Figure 10:**
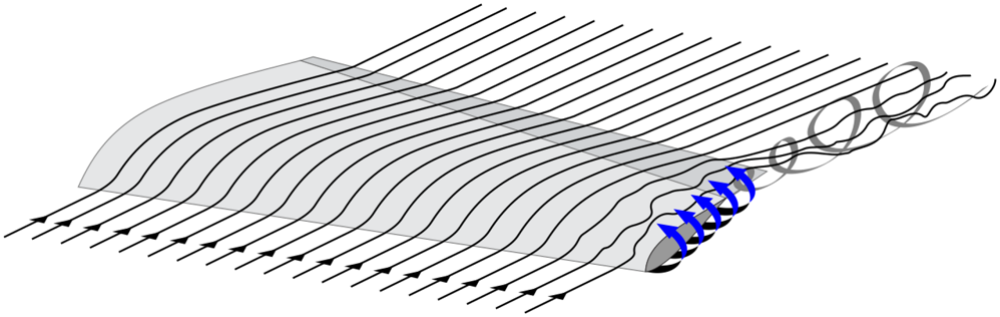
Pressure distribution on the wing surface disturbance by the flow field on the wingtip.

Hence, we have chosen a flying kite with a $\Delta$ shape geometry, which provides a low drag coefficient with almost the entire structure generating lift in comparison to a traditional aircraft. Nonetheless, lift may be compromised by the induced drag effect, which interferes in the distribution of pressure of the wing surface. This is a well known problem in aerodynamics, caused by the pressure difference on the region between the wing extraction and the soffit. This makes the wind that reaches the top of the wing to generate vortexes on the wingtip disturbing the flow field as seen in Fig. 10.

**Figure 11:**
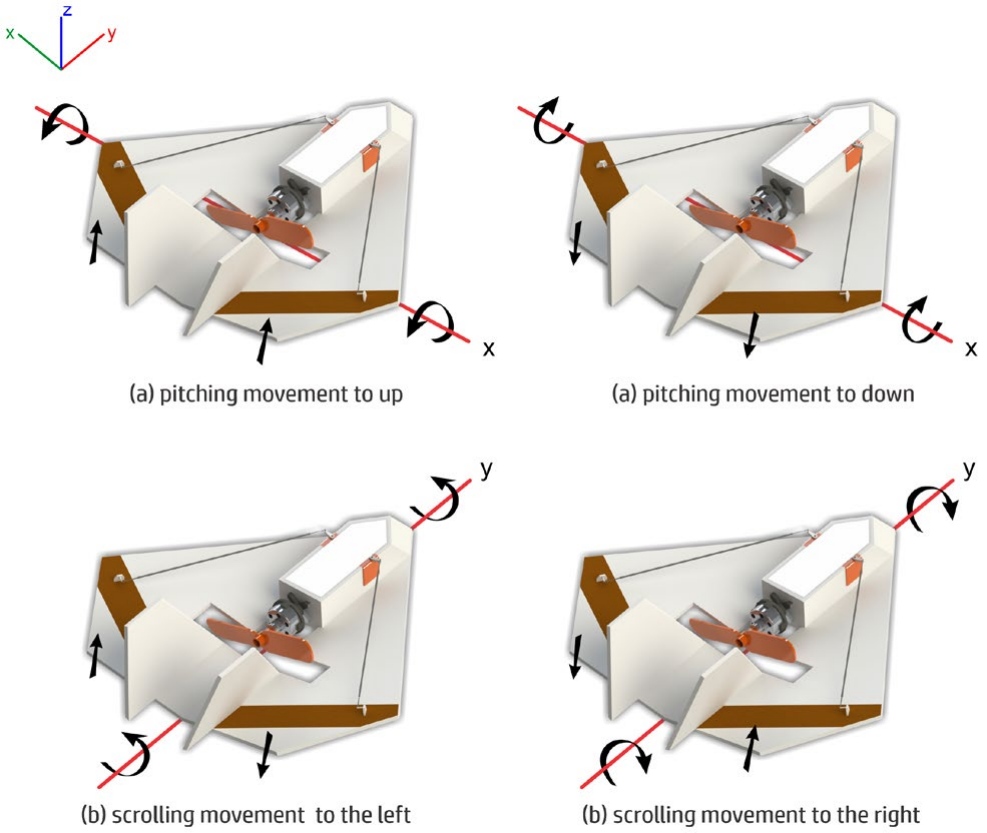
Movements that the MicroBrosh motion control system can perform.

To prevent the formation of vortexes, we make a cut in each wing panel as seen in Fig. 9. Besides preventing the formation of the vortexes during flight, this increases the induced drag and do not compromise the efficiency of the control surfaces. Next step is to define a simple yet efficient system for control of motion. For that, we use a elevon configuration as it only uses two control surfaces driven by two servomotors. The aircraft can perform scrolling movements and panting with mixed movements of the elevons. The pitch is performed when the control surfaces move both in the same direction as seen in part (a) of Fig. 11. Scrolling to left or to right is performed by moving the control surfaces in opposite directions, as seen in (b).

**Figure 12:**
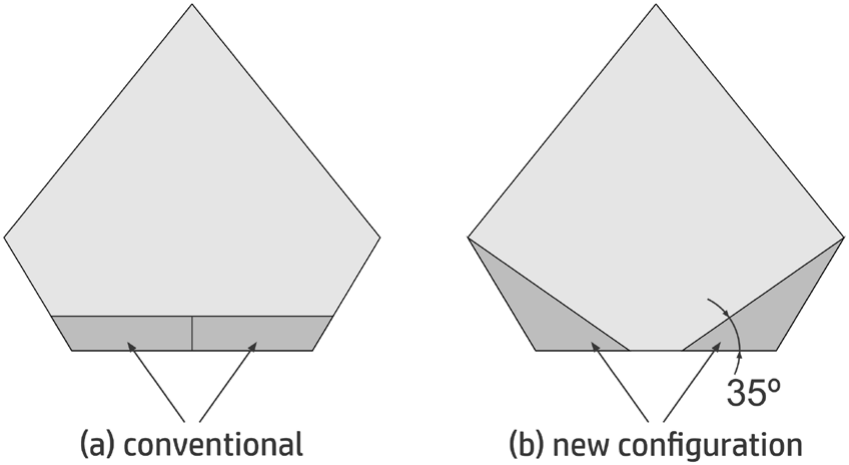
Conventional configuration (**a**) with control surfaces modified with an angle of 35$^{\circ }$ (**b**).

Notice that the control surfaces provide some lift as they are part of the wing. Thus, the aircraft loses some lift when the control system performs scrolling movement. A correction for that can be done with a little of pitching. Also, we provide a modification on the control surfaces in order to prevent complete loss of lift in the case of scrolling. These conventional control surfaces shown in Fig. 12a have a new configuration, with a 35 $^{circ}$ angle, as shown in Fig. 12b. Scrolling and pitching are preformed in a mixed way, which provides a partially automatic correction. This makes the remote pilot controls even easier.

**Figure 13:**
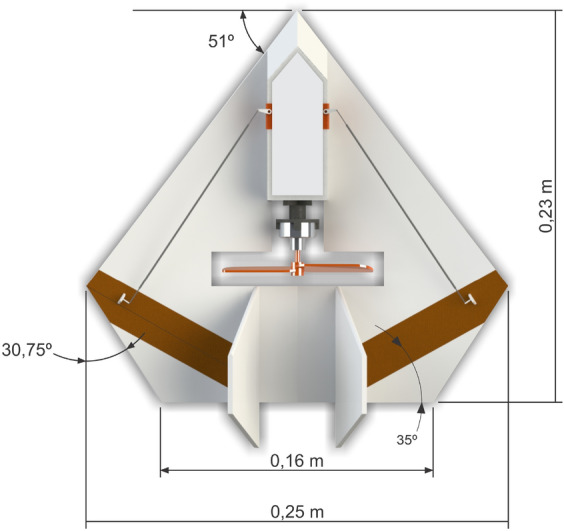
MicroBrosh conceptual project in top profile view.

**Figure 14:**
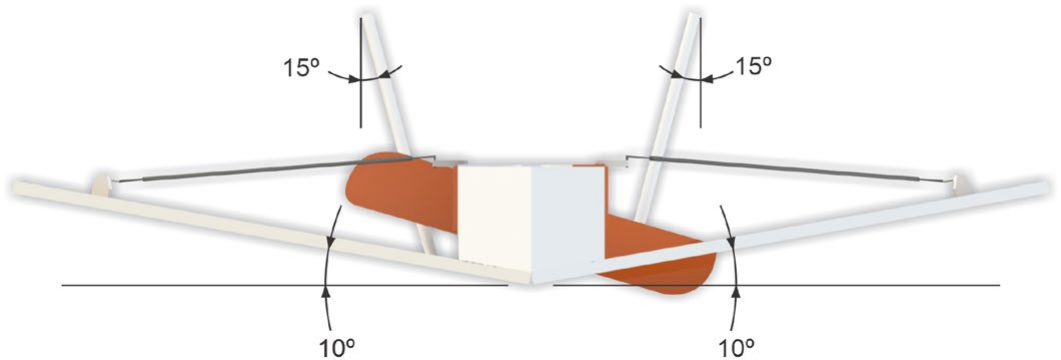
MicroBrosh conceptual project in back profile view.

Longitudinal stability during flight is provided by way of using two vertical stabilizers with inclination of 15$^{\circ }$. In addition, we add a positive 10$^{\circ }$ dihedral to the wing to provide lateral stability. The propeller of the chosen pusher configuration power-train in this version is located close to the center of gravity, as seen in Fig. 13, however with the option of being at the tail of the aircraft in the next version. Here, the box seen after the propeller extending to the aircraft front has the electronic system embarked, which, besides the motor for the propeller has the two servomotors for the motion of the control surfaces and possibly the automatic pilot. The next version also incorporates the camera. Front and back views of our initial conceptual project with its main measures and angles are seen in Figs. 13 and 14, respectively.

**Figure 15:**
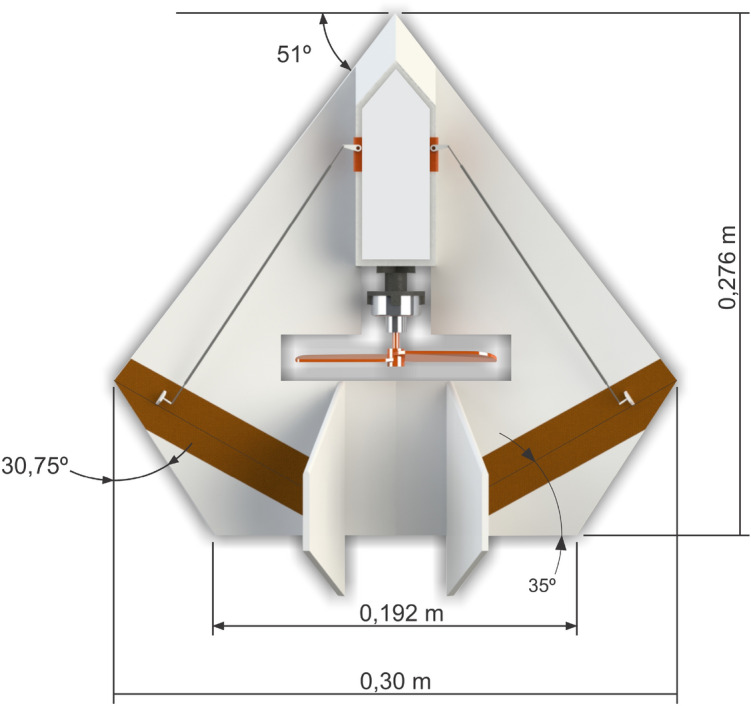
Final prototype measures.

Hence, the inclusion of more embarked electronics in our final prototype, especially the automatic pilot and batteries, has increased the total aircraft weight, thus increasing the necessary lift. So, for the version with autopilot and camera with video recording, we have to perform some small modifications in the UAV structure in order to support them. Mainly, we keep the basic design while proportionally increase the aircraft dimensions, adding an aerodynamic profile and removing the dihedral. These modifications of this second version of our final prototype can be seen in Fig. 15, with the propeller still in the center. However, in order to support more payload and keeping the center of gravity, we have the option of putting the propeller in the back of the wing. Actually, this is done to support autopilot and camera together, as will be seen further, without compromising safe requirements.

### Prototype construction

**Figure 16:**
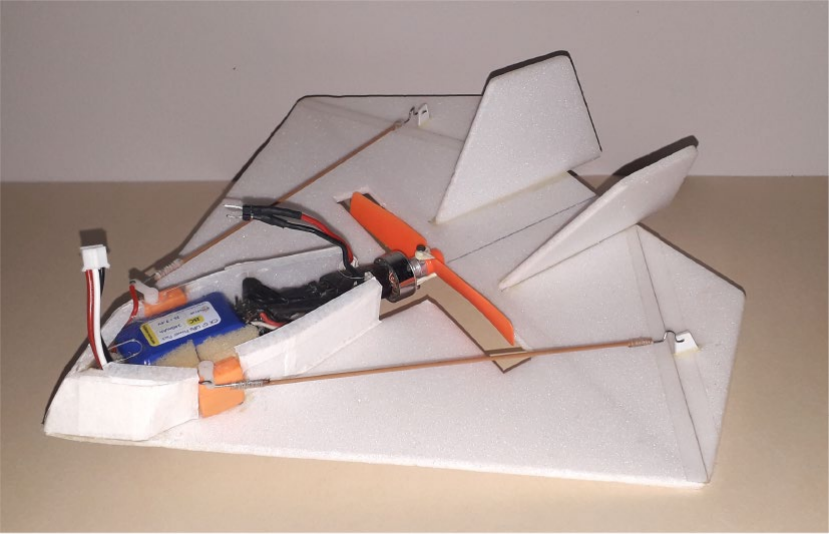
Process of construction and assembly of the final prototype with propeller at its center.

We notice that a series of 5 previous prototypes with closely the same configuration have been built and extensively tested, previously to the final one depicted in detail here, which has two versions. For the first version, we manually corded the wing using a extruded polystyrene flat plate with 4 mm. We use epoxy resin to glue the electronics compartment and the vertical stabilizers. We make the control surfaces articulation arms and the fixing wall for the propeller assembly using a plate of polystyrene with 2 mm. The servomotors are connected to the control surfaces articulation arm with 0.5 mm fiberglass rods that are made with steel wire terminals. Also, we use transparent polystyrene with 0.5 mm plate for closing the compartment and *3M* adhesive tape *Blenderm* model for the hinge function of the control surfaces. The minimal final weight is 62 g with one battery and remote control, which is within the primary requirements of the project. However, as said, in order to accommodate the autopilot and camera link, a modification should be done. It should be added another 0.034 kg for the autopilot, besides the camera system and a second battery, totalling 0.145 kg. The two batteries are 0.038 kg. The final resulting prototype with central propeller can be seen in Fig. 16, without the camera.

**Figure 17:**
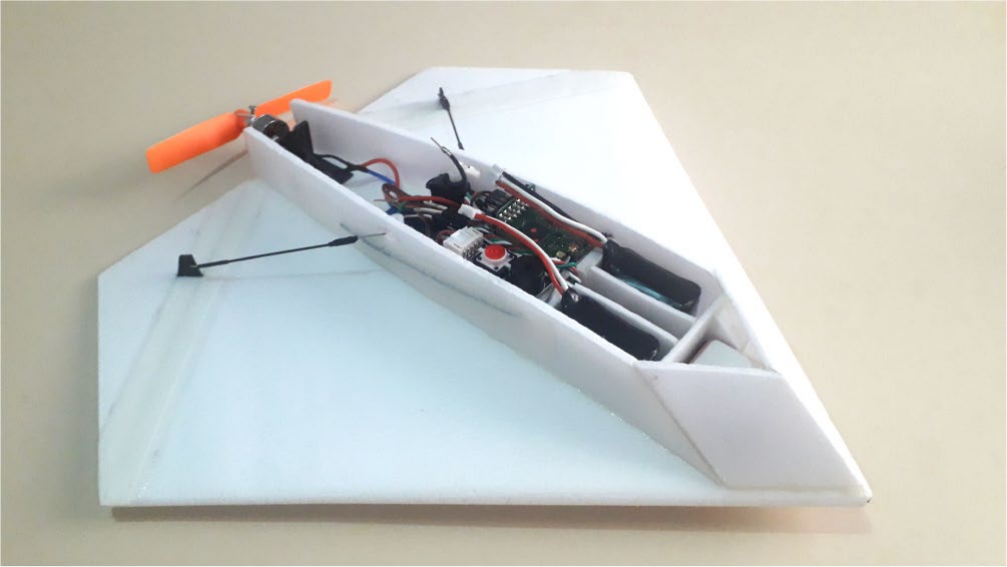
Final prototype with propeller at the tail.

**Figure 18:**
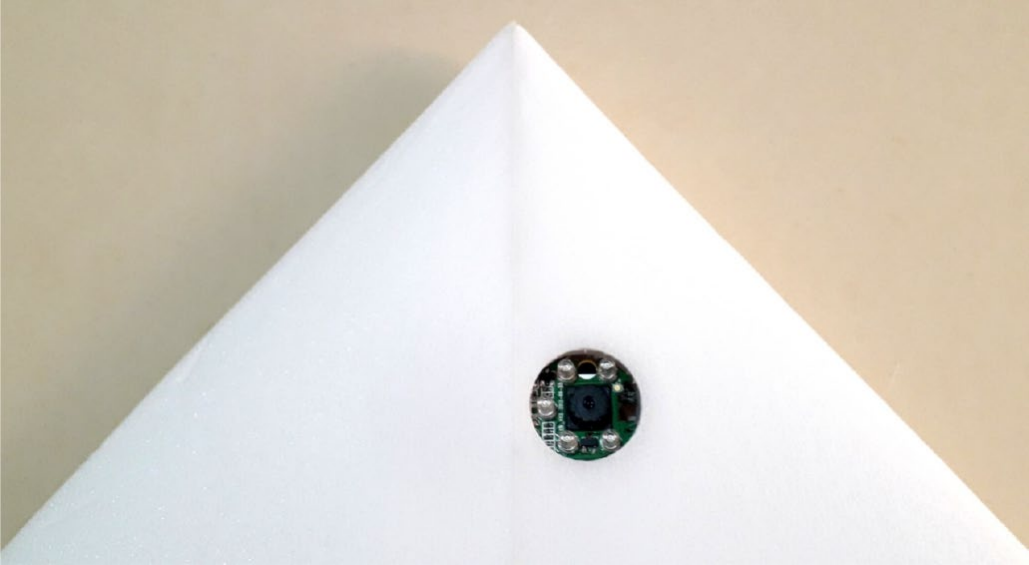
Camera mounted in the wing fuselage (with down view).

The final version with automatic pilot configuration and with back propeller and camera can be seen in Fig. 17. The fuselage that accommodates the embarked components (including camera card recording hardware) is manufactured with a extruded polypropylene foam plate with 3 mm. We added a rail-shaped chassis for embarking the autopilot. The rail is added in order to vary its position and make it easier to adjust the center of gravity. With the fuselage complete, the components are installed, coming up with the final layout shown in Fig. 17. The mounted camera in the aircraft is seen in Fig. 18, from a down view.

### Embarked electronics and control system

**Figure 19:**
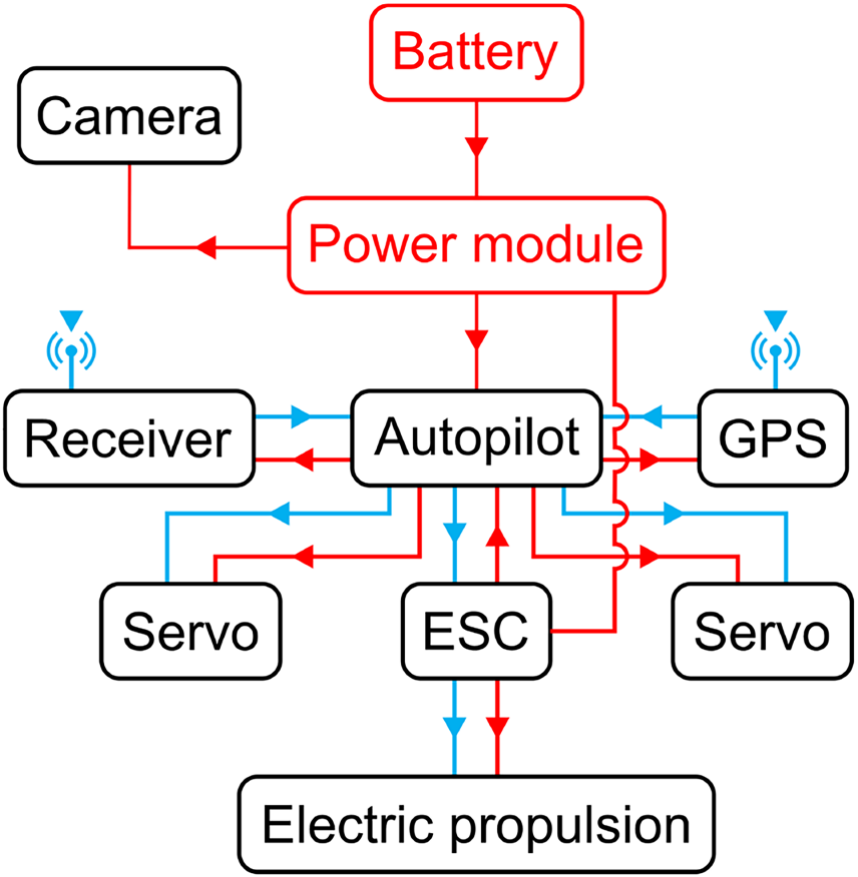
Embarked components for autonomous flight configuration including autopilot and camera.

We aim to use a minimum of electrical and electronic components in the design of the propulsion and control systems, initially coming up with the basic electronic architecture that is seen in Fig. 19. A simple configuration allows the aircraft to be operated both using the autopilot and also by remote control. The version without autopilot uses a Spektrum transmitter model DX7 2.4 GHz. This radio works with a receiver *Orange*, model DSM2 Atmega168PA, which has an automatic gyro stabilization system with adjustable gains that can be switched on and off using a selector on the transmitter, even during the flight. We use a Traxxas model 6348 nano-digital servomotor to control the surfaces motion. The propeller is activated by a power-train set with 2000 kv, three-phase, which uses a Brushless motor from RC-Hobby. The propeller is a GWS model EP-4025. A Controller (ESC) from Hobbyking, model HK-10A, controls the power-train. A single Li-Po 2S with 7.4 v and 240 mAh battery model Hyperion G3 weighting 0.015 kg provides the necessary power for the whole embarked system of this first version.

**Figure 20:**
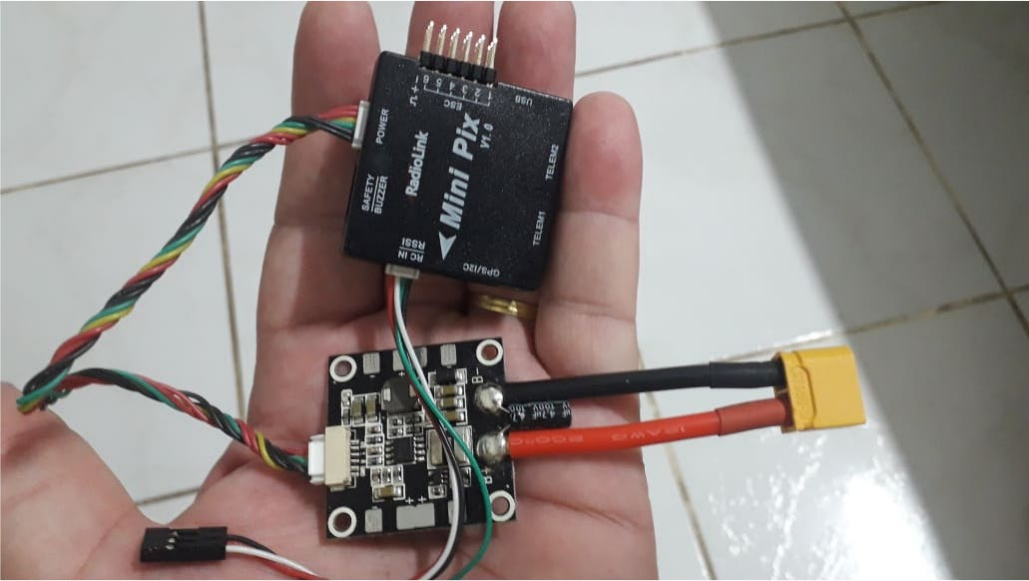
Autopilot embarked in the prototype.

In order to embark the autopilot shown in Fig. 20, which weights 0.034 kg, and extra battery, we define a new version configuration as shown in Fig. 19. This new version has a controller board brand Radiolink model Mini Pix V 1.0, aided by a GPS TS100 Mini M8N, a brushless motor with 3800kv, Hyperion LiPo brand battery model G7 SV Si-Graphene with two 7.4V with 300 mAh capacity and 50C discharge rate. Notice the radio control system that is replaced by a brand Futaba model T8J 2.4 Ghz transmitter and a model R2008SB receiver. This exchange aims to use the serial data system between the receiver and the autopilot, which ensures greater accuracy than using PWM. The servos responsible for activating the control surfaces remain the same as in the radio controlled model, the components are detailed in Table 2. Hence, we can now perform autonomous flight, which can be done using just by configuring it using the Mission Planner software as in our previous work ^53^. The *Mission Planner* software is open source.

**Table 2:**
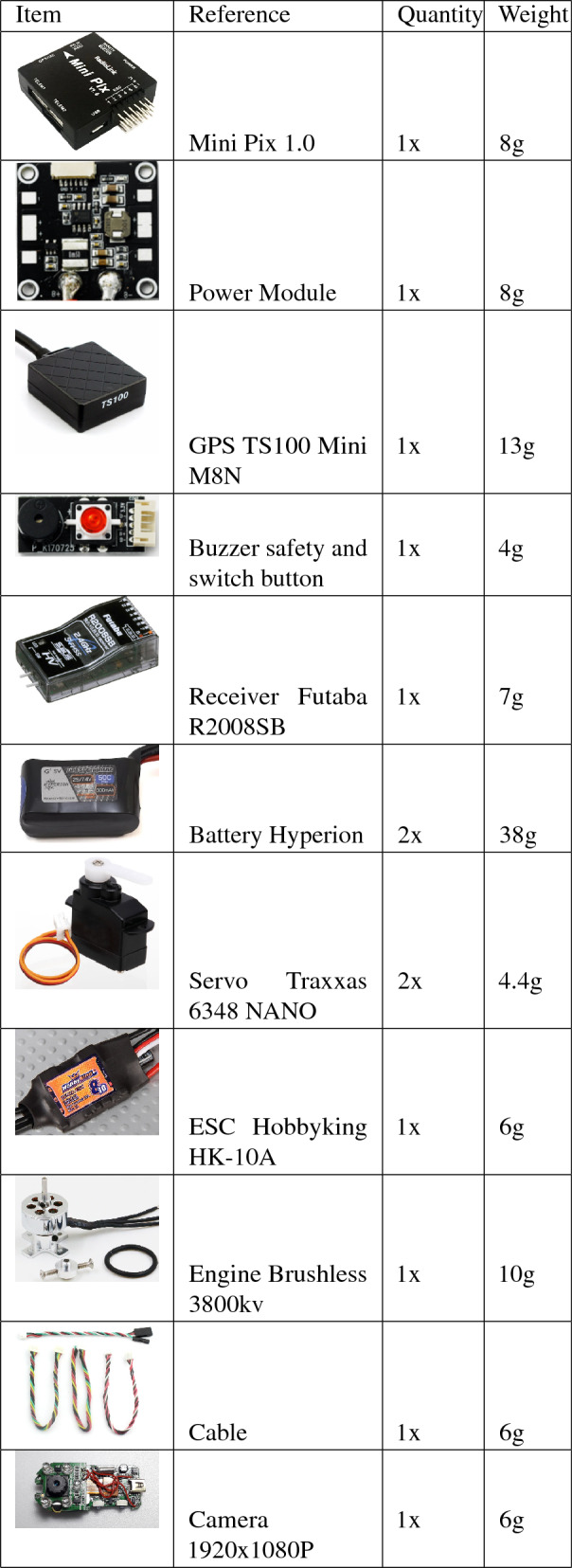
Electric and electronic components used in the development of the MicroBrosh UAV final prototype.

## Experimental setup

We have performed several types of experiments in this work in order to deploy our final prototype UAV totally operational. At first, as explained above, in an empirical finding, we have built and tested several prototypes (9 in total, with more than 15 aircrafts overall) to determine the one that has the better responses to our requirements. The practical tests (flights) that are reported first in this Section are done to determine wind sustainability and the smooth control of them. After coming up with prototype number 9, which was the best in all tests, we go to other detailed tests that we report here, in order to see its several aspects. At first, we perform bench tests in order to calculate the aerodynamic variables and we also perform thrust analysis. Then, we have done indoor fight tests in controlled conditions and also several outdoor flights with severe wind conditions. In these tests we used the parameters that were obtained in the bench tests to feed the system for the field flight testing conditions. After all, we develop the last version of our prototype 9, which is a little bigger (wingspan of 0.30 m) which supports autonomous flight and camera recording. Hence, in this Section, we describe all of these tests, the first ones with some of the prototypes that have behaved better and we also show experiments done in the aircrafts of the two versions of our final prototype.

The aerodynamic analysis for the design of an aircraft is a fundamental part for its efficiency, so it was inserted for the development of the last prototype. The analysis of the NACA 0006, EPPLER EA 8(-1)-006 and the Gottingen 443 profiles and the choice of the best option was tested on this prototype model, which enhanced the shape of last aircraft produced (prototype 9, final).

As well as aerodynamics, the performance of the powertrain also directly interferes with the efficiency of the aircraft, each type of propeller will provide unique characteristics during the flight, varying the speed, thrust and power available for each percentage of acceleration, consumption of the energy matrix and rotations per minute. To verify this, we developed and used the test bench with all the necessary components for the detailed analysis of the powertrain, resulting in important information for the selection of the set used in the last prototype.

### First flight experiments for finding of the right prototype model

**Figure 21:**
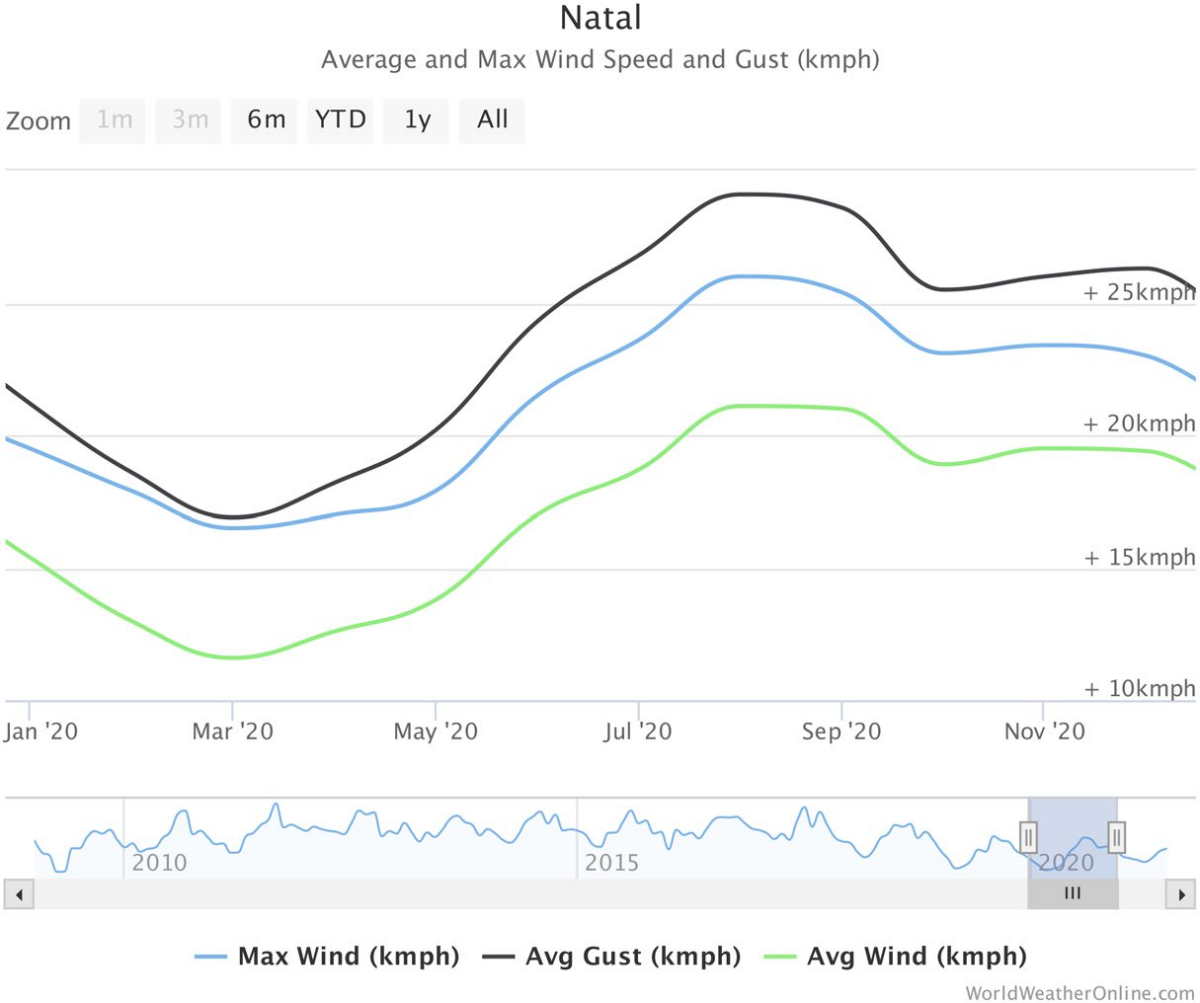
Max and average wind speed and wind gust during the year of 2020 for Natal, Brazil.

As introduced above, in order to choose the right aircraft model, we have initially done a series of experimental flights with several, different fixed wing based aircraft prototypes that we have constructed, varying from traditional airplanes to flying wings. For each built prototype, in a total of 9, we initially perform indoor flight to test stability and other issues and, once it works, they go straight to outdoor tests. Our experiments happened in Natal, northeast of Brazil which is known to have extreme wind conditions, with wind velocity varying from 12 km/h to 22 km/h and wind gusts varying from 18 km/h to 29 km/h, as it can be seen in Fig. 21. Most severe wind conditions are experimented in Natal from May to the beginning of September. The wind direction is almost the same during these months, which is mostly of the time from southeast. So, a planning on the flights for several weeks or months can be done without need of re-planning due to these repeatable (and worst) conditions during the period. The main purpose is to test engine/battery/propeller assembly performance in flight as well as lift/stability and pitch and swing control commands. In the next we report tests and results for the best models.

#### Prototype 4

A flight remotely controlled by radio is held in an indoor gym with a slight presence of wind. Manual launch. The prototype does not present stability in flight, showing little support and lack of motor traction. The response to the pitching movement is lower than expected, besides the movements in the longitudinal axis produced by the ailerons are satisfactory in both directions of rotation. We performed no yaw tests due to the option of removing the rudder in order to reduce the weight of the prototype.

#### Prototype 5

This prototype is configured with elevon standard, mixed by radio control. Its initial flight conditions are outdoor remotely controlled radio flight, carried out in a grass field with strong presence of wind, with manual launch. The prototype does not show stability in the flight, so we have no conclusive data on pitching and turning movements, the lack of motor traction presented in the prototype 4 becomes better with this one.

#### Prototype 6

Prototype 6 has a wingspan of 0.40 m, which improves stability and sustainability, so this aircraft tests are done also to obtain information on the efficiency of control commands of heaves and turns. The flight conditions are as outdoor remotely controlled radio flight, carried out in a grass field with strong presence of wind with manual launch. This prototype presents excellent stability during flight, acceptable lift with acceleration at 30% of capacity, pitch response within expectations, excellent turn response in both directions of rotation, excellent climb rate, maintaining stable flight and acceptable response of the control commands during the maneuver.

#### Prototype 8

With prototype 8, we test the performance of the engine/propeller/aircraft assembly with greater ability to reduce the torque effect, as well as lift/stability and the pitch and swing control commands with a wing profile close to a smooth plate. We perform outdoor remotely controlled radio flights, carried out in a grass field with strong presence of wind, with manual launch. This prototype presents excellent stability in the manual launch flight using the engine, but it does not present stability in the radio-controlled flight using the engine, even though we considered its wing profile parameters excellent data for a new aircraft configuration.

#### Prototype 9

This prototype has presented excellent stability in the manual launch flight without the use of the engine. The objective of this manual launch is to test the performance of the engine/propeller/aircraft assembly with greater ability to reduce the torque effect, as well as lift/stability and the pitch and swing control commands with a wing profile using a smooth plate with a slit edge, in rounded attack. For that, we perform outdoor remotely controlled radio flights, carried out in a grass field with strong presence of wind, using manual launch. It also presents excellent stability in the radio-controlled flight with the use of the engine, where the torque effect is considerably reduced compared to the previous aircraft. Its first flight reaches a 25 min of duration. Complementary flights with better weather conditions reaches 35 min of duration and the control of the aircraft, even in strong wind conditions, proved to be very satisfactory.

### Simulation tests for aerodynamics

**Figure 22:**
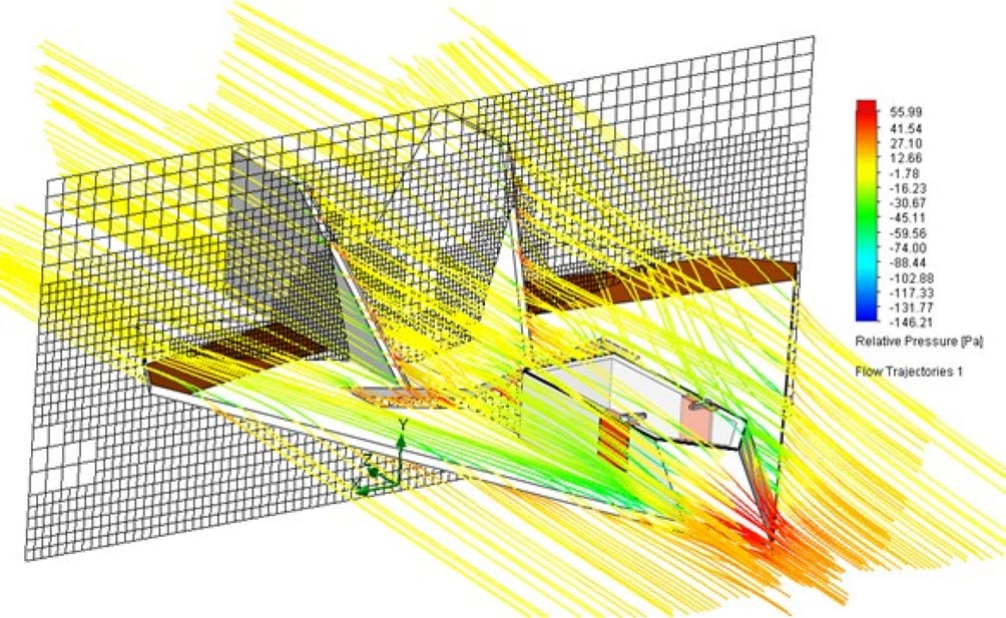
Flow trajectories with relative pressures.

**Figure 23:**
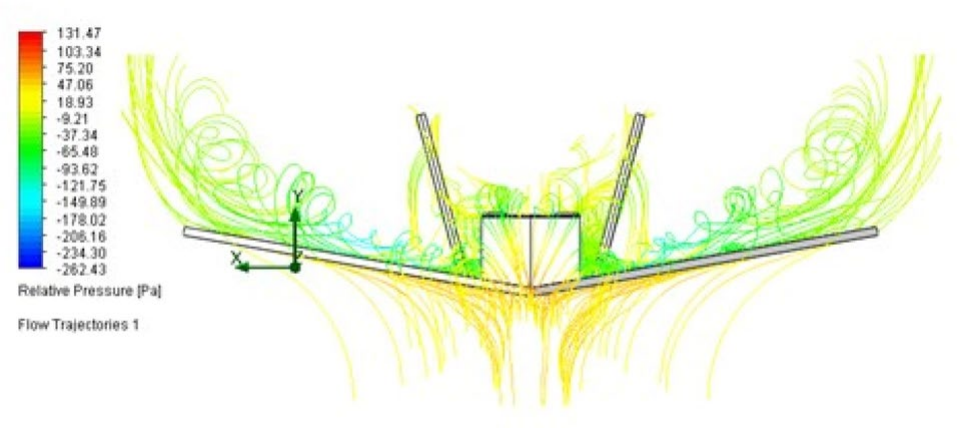
Flow trajectories with relative pressures.

We use 4mm flat plates without rounded edges for making the initial aerodynamic for the wing and vertical stabilizers, without a profile definition yet. This kind of material offers easy of manufacturing, both manual and industrialized. Hence, we should obtain the coefficients of support and verify the possibility of vortexes generation on the aircraft wing. For that, we perform an analysis using computational fluid dynamics (CFD) simulation. These tests are made using the software Solid-Works. we used the input value of 12.5 m/s for the flow speed, with variations of 2$^\circ$, 5$^{\circ }$ and 10$^{\circ }$ for the angle of attack, as seen in Table 3. The maximum possible number of particles is defined automatically. The resulting flow simulations are shown in Figs. 22 and 23, for slanted and back profiles.

**Table 3:** CFD results at 2$^\circ$,5$^{\circ }$ and 10$^{\circ }$ angles of attack.

$\alpha$	$C_l$
2$^{\circ }$	0.100
5$^{\circ }$	0.229
10$^{\circ }$	0.584

### Thrust analysis

**Figure 24:**
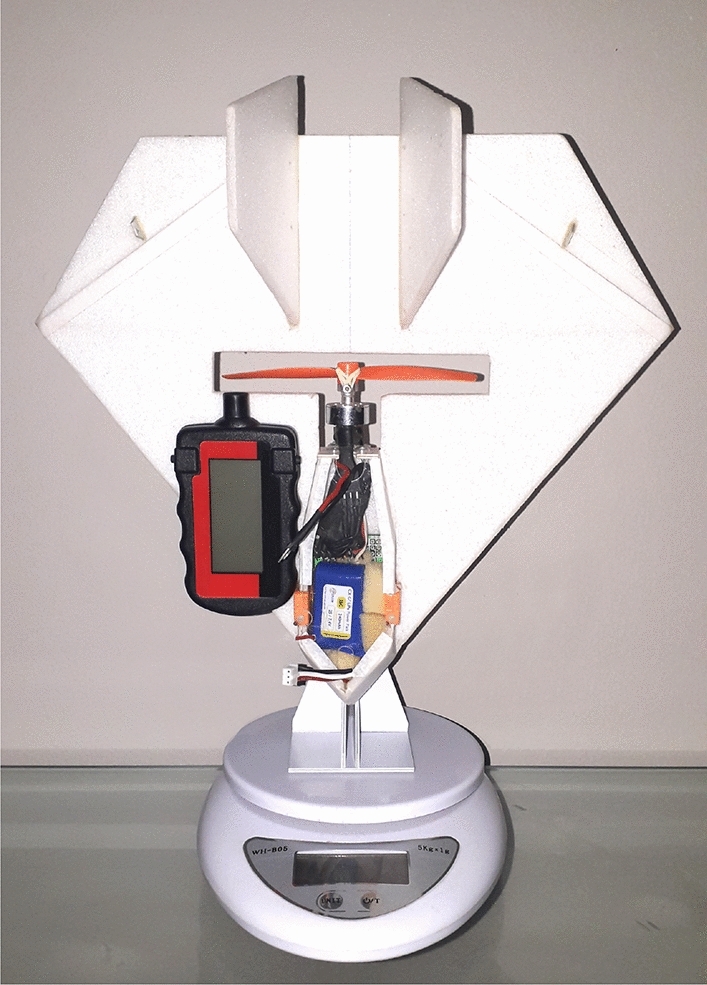
Digital scale with high-precision and digital spin counter HCAP0400 used for static thrust determination.

**Figure 25:**
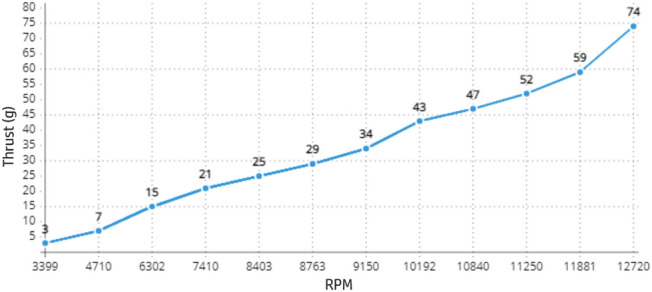
Graphic of the static thrust in 12 RPM rates. A maximum of 74 g for a rotation per minute rate of 12720 (RPM) is achieved.

With initial aerodynamics defined, we perform initial experiments with the first version without autopilot, previous to profiles definition, for analyzing the static thrust generated by the power-train that is responsible for pushing the aircraft forward, thus generating the necessary lift. For that, we use a simple apparatus with a digital spin counter (model HCAP0400) and a high precision digital scale as seen Fig. 24. Besides already installed in the aircraft, we analyze the power-train of this initial version separately in order to account for the losses that are eventually generated due to some interference of the the aircraft. A graphical analysis of this experiment can be seen in Fig. 25, where we show the computed acceleration in 12 points. The power-train of this configuration goes to 12,720 rotations per minute (RPM) giving a maximum static thrust of 74 g.

### Profile selection

**Figure 26:**
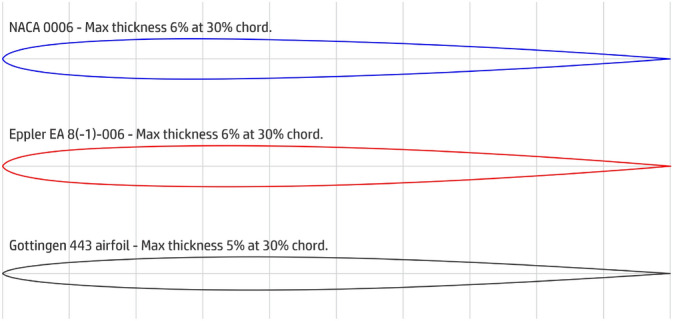
Profiles tested for the final prototype.

**Figure 27:**
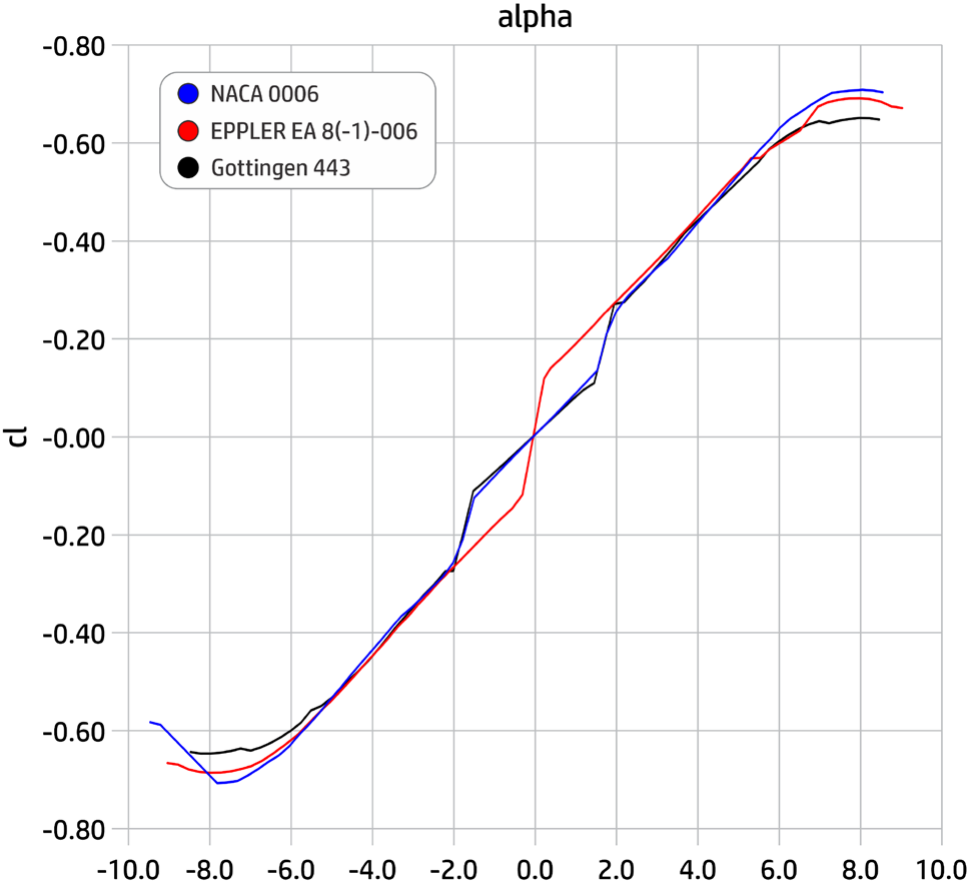
Support (lift) characteristic curves (cl).

**Figure 28:**
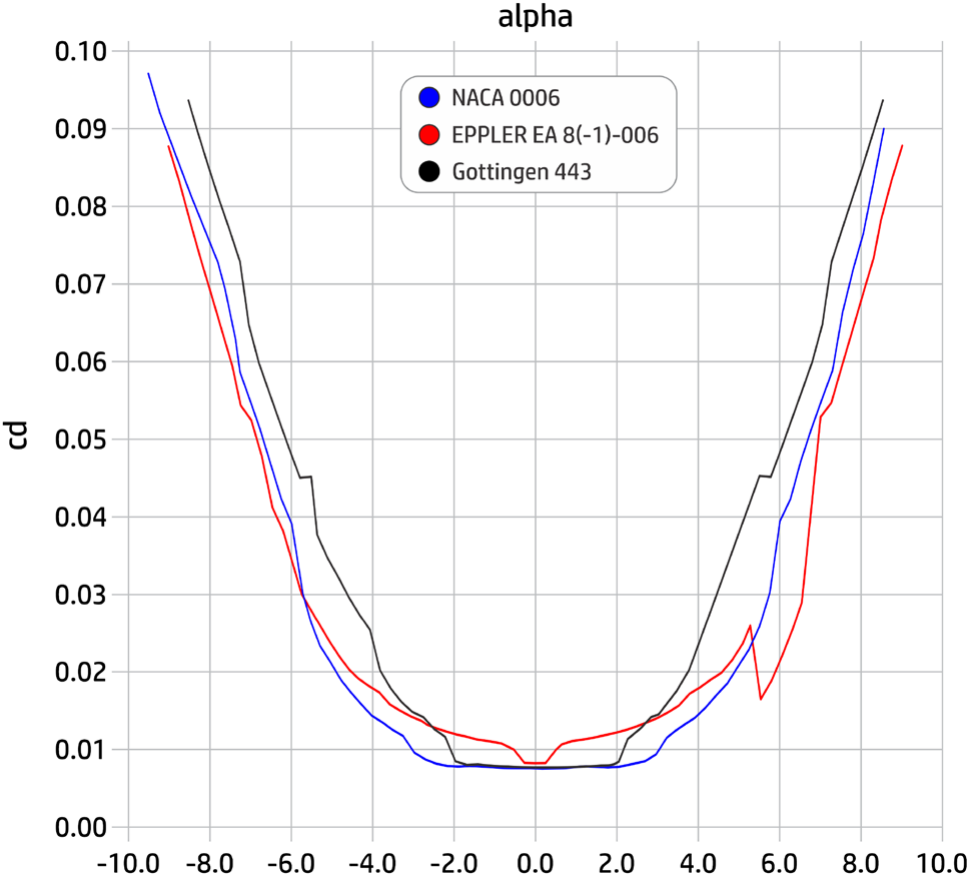
Drag characteristic curves (cd).

With the aircraft a little bigger, we decided to use a profile in order to enhance aerodynamics. Between the available profiles, we select three, here, for been verified, the NACA 0006, EPPLER EA 8(-1)-006 and the Gottingen 443, seen in Fig. 26, from which we have to choose the best one. For that, we use the Xfoil 6.96 software to generate the data for the graphs of the characteristic curves of lift (cl) and drag (cd) against slope variation (attack angle $\alpha$). Resulting curves for the lift and drag coefficients are shown in Figs. 27 and 28. From analyzing these graphics, we finally select the NACA 0006 profile due to its bigger lift and its lower drag coefficients.

### Choosing a propeller

**Figure 29:**
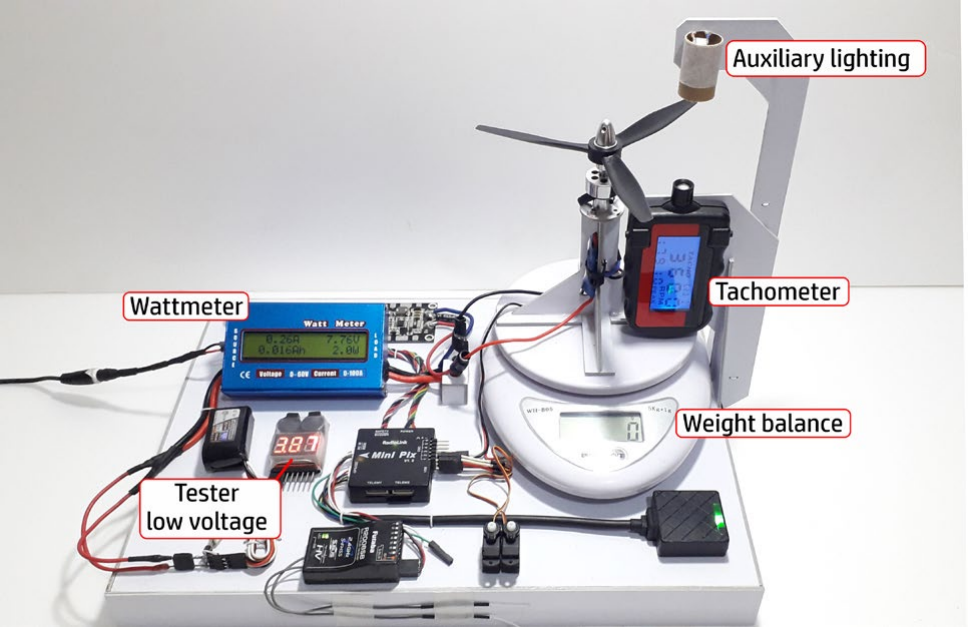
Bench test platform for determining propellers parameters.

The ability to perform a flight with efficacy and the aircraft’s performance characteristics are directly linked to the powertrain. Thus, another important step is the choice of the propeller, since there is a wide variety of possibilities in terms of material, diameter, pitch and number of blades. For that, we developed a test bench consisting of a wattmeter, a tachometer model HCAP0400, a high precision digital scale, and a low voltage meter to check the energy consumption in each battery cell, mainly to avoid them blow up during tests. Because the same battery is responsible for powering the powertrain and on-board electronics, we installed all the components of the aircraft on the bench in order to simulate the actual consumption during the flight. The apparatus, with the installed components, can be seen in the Fig. 29.

**Figure 30:**
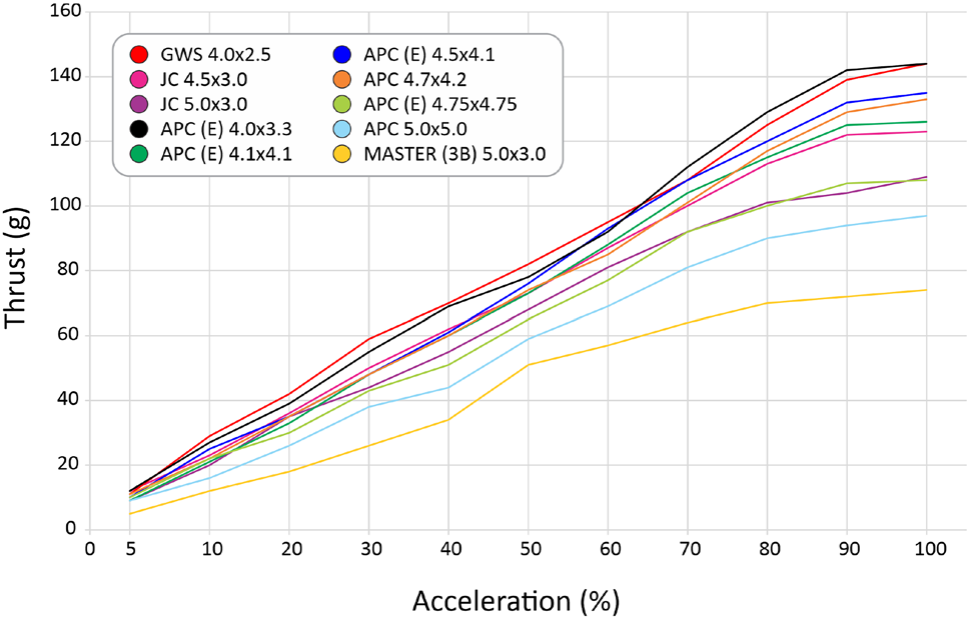
Curves for the thrust generated by propellers as a function of acceleration.

**Figure 31:**
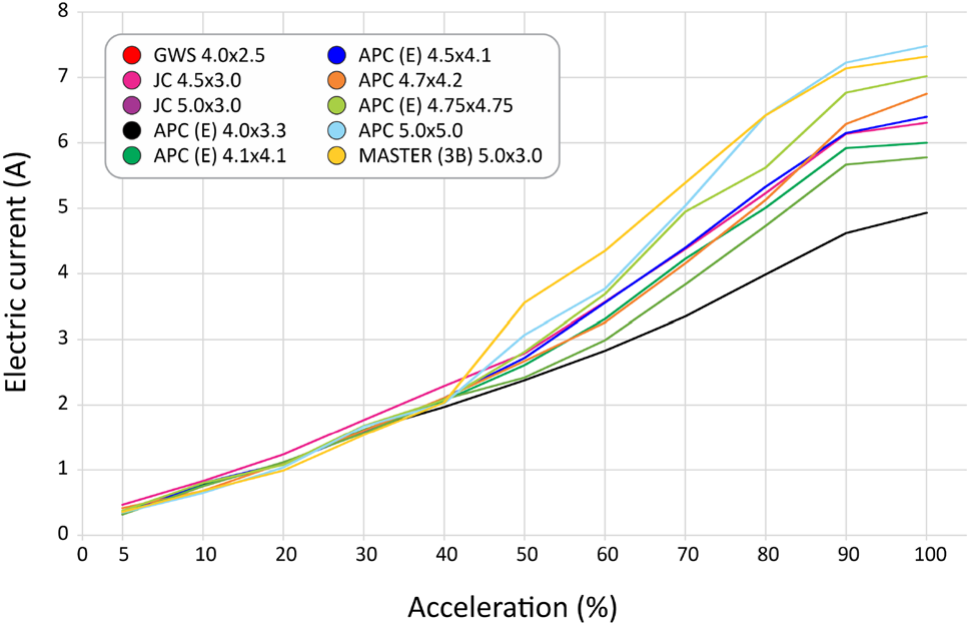
Curves of the electrical current consumed with the use of each propeller as a function of acceleration.

**Figure 32:**
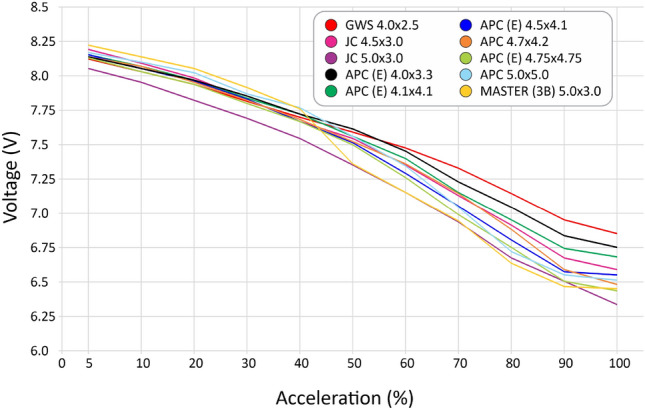
Voltage curves available by the battery with the use of each propeller as a function of acceleration.

**Figure 33:**
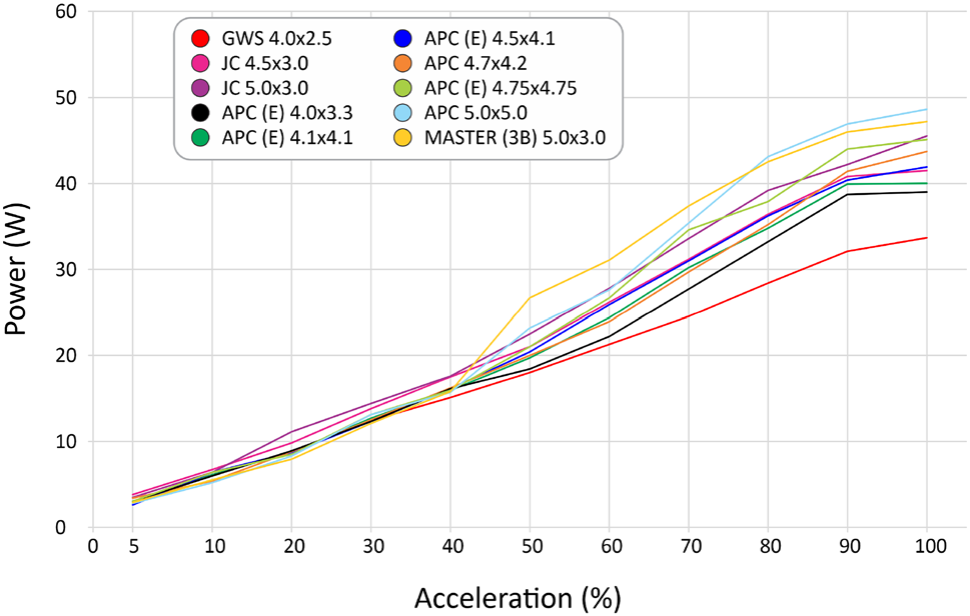
Curves of electrical power consumed with the use of each propeller as a function of acceleration.

**Figure 34:**
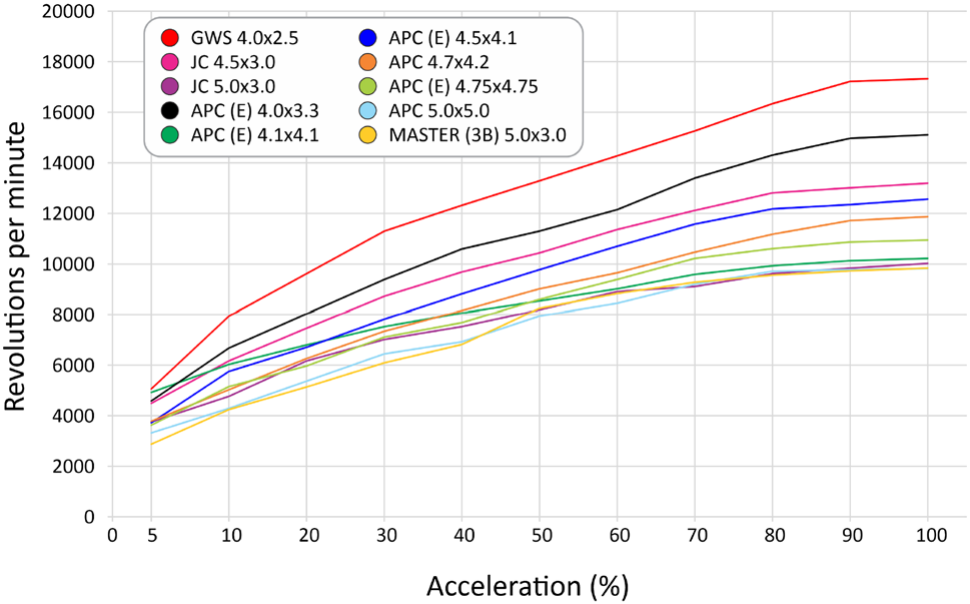
Rotation curves per minute of each propeller as a function of acceleration.

We selected 10 different, most used propellers including materials as ABS, wood, polyamid and using different profiles for some of them, as seen in Table 4. After the tests, we computed the curves for thrust (Fig. 30), current (Fig. 31), available voltage (Fig. 32), consumption (Fig. 33) and revolutions per minute (Fig. 34), all as a function of acceleration. After these tests, we have chosen the GWS because it has the better thrust with less consumption.

**Table 4:**
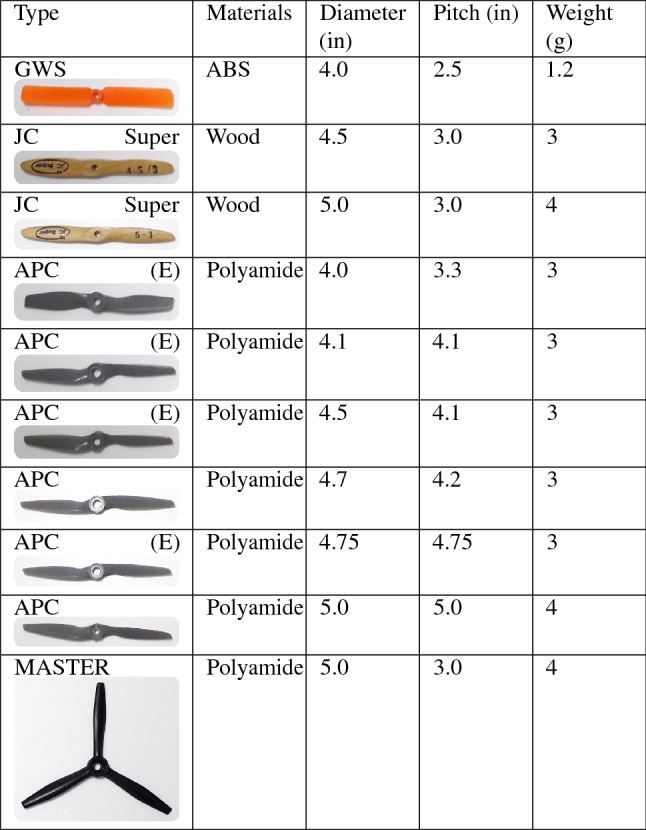
Propellers chosen for the tests.

### Flight tests for prototype 9

We performed the first (controlled) flight with our first version in a gym without stabilization at principle, just to adjust the surfaces for controlling straight flight and also to verify the positioning of the gravity center. These flights were done Without using remote control, with manual launching with the aircraft at a 40% of the total possible acceleration. We figured out that the UAV trends to the left, besides we have aligned the control surfaces. This is mainly observed when increasing acceleration certainly due to the effect of torque that is generated by the aircraft propeller. When it reaches a 60% of acceleration, it starts turning around, in the *y* axis both in straight flight mode and in curves to the left. This problem gets worst in higher acceleration, making it impossible to control the aircraft at some point, let’s say with 100% of acceleration.

Next, we performed tests using the stabilization system, with 25% on the performance scale at start. The UAV got support with acceleration in approximately 40% similarly to the previous flights, with the torque effect reduced. However it presents a small trend to the left and still performs turns around the *y* axis. However, we could execute flights where the acceleration is 100% with the stabilization system at 50%. This has eliminated the torque effect problem. we also performed other tests in which the stabilization is at 60% and 75%. However, we noticed an interference in the commands sent by the remote control after some 50% of stabilization. This has completed the indoor tests.

Hence, we start the outdoor tests with the wind presenting intermittent conditions. As presented above in Fig. 21, Natal is a place of severe wind conditions, with wind gusts going up to 30 km/h. Thus, we start the flights manually and with 50% of stabilization. In a first test, we could keep the control of the MicroBrosh, similarly to indoor tests done. We get the lift supporting the aircraft in down wind situation with about 40% of acceleration. When the flight is against wind, this can be even much smaller, with acceleration going down to some 5%. Because of the worst wind conditions that causes subtle changes on the MicroBrosh direction in several instants of the flight, the aircraft looses stability. So, we correct this after increasing the stabilization rate to above 55%.

**Figure 35:**
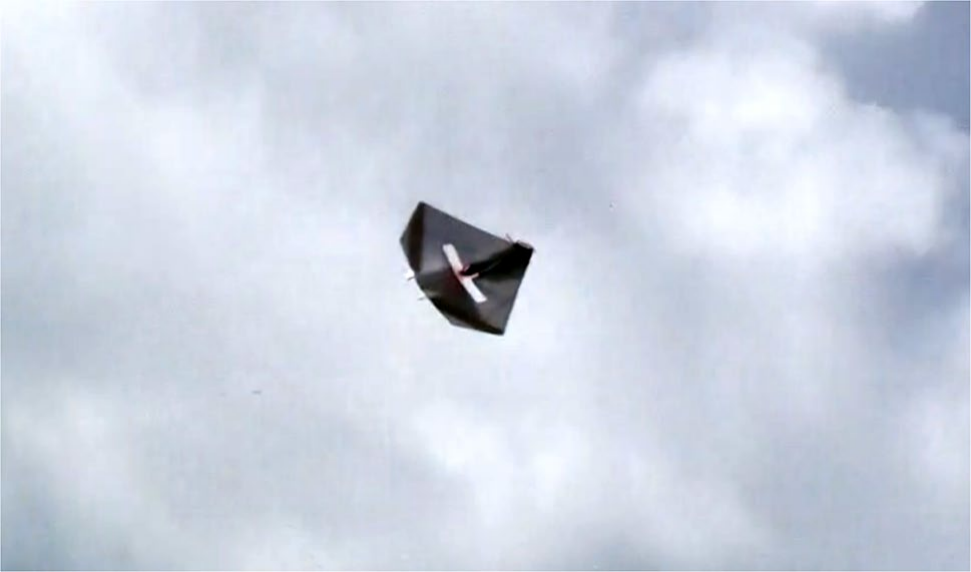
Picture from experimental flight performed (video with about 4 min of flight duration available at https://youtu.be/5nNde87ctaY).

Thereafter, we have adjusted the aircraft’s behavior and it could perform a series of flights without problems. Actually, in one of these experiments for demonstrating the flight conditions and smoothness, we record a video showing the behavior of the UAV ^67^. A frame extracted from the video can be seen in Fig. 35. This flight duration is about 4 min and we use a one battery configuration here, with enough energy remaining at the end for another flight.

## Discussions

As said, the field flights were performed outdoor with the wind presenting very extreme conditions as it can be observed in the above video. Flight duration is discussed below. Nonetheless, besides the interesting advantages of our proposal, there are some limitations that we also wish to point out in this Section, next to flight endurance.

### Flight endurance

We notice that the above flight configuration has been enhanced with a two batteries in our final version, so we expect that the time of flight gets extended to some 30–40 min. This two batteries option also makes it possible to have a camera embarked, as seen above in Fig. 18. With that we estimate that the UAV is able to perform a flight with a distance of about 20 km, with the last version (Fig. 17). Just for comparison, ours is smaller and with less weight than the state of the art but the Widon ^59^, and with the best speed rate ^62,63^. Outdoor tests have been stopped due to the pandemic, however, we still have space for enhancements that we believe will improve yet more these performance items. New developments on the mechanical system and using more batteries will produce better results.

### Limitations

One first aspect that has been improved in the last version, however, that needs outdoor tests, is the autopilot. It has been embarked and we got it running just recently, so it has not been tested outside, just some inside tests have been done. Our control model uses a generic algorithm, which comes with firmware and that is provided by the Mission Planner software ^68^. All of the flights were performed remotely controlled, however, using the software for stability reasons. Nonetheless, we have to wait for better conditions of the Covid-19 pandemic in order to perform more tests on this issue. To date, the currently constructed version has a verified RC model aircraft with stabilizer, and all equipment for supporting the autonomous UAV capability is already embarked.

We now have video capture capability, which is stored in a flash card. This item should also be enhanced with the possibility of video transmission, thus allowing for some user to take decisions on-flight, in real-time. This can be easily incorporated in our system, with nowadays several available products. After this, our Micro-Brosh UAV can be made available for missions in which this straight control is necessary. Also, including BVLOS capability and testing it is another feature that should be worked on. Finally, we remark the wind conditions in Natal, Brazil Northeast, which has velocities varying from 10 km/h to 20 km/h with gusts ranging from 17 km/h to 30 km/h. Our final version has been stressed in these conditions and has shown stability, as can be seen from our results (and verified in the video). Further, we should stress features as wind penetration and stability in stronger winds, that are not observed here, for example near (or inside) a hurricane conditions.

## Concluding remarks

We introduced current efforts towards the development of a micro UAV named Micro-Brosh with less than 0.2 kg weight and 0.3 m wingspan, thus offering low risk of operation to installations and persons. Our proposal includes the architectural design of the UAV with a wing type geometry, showing flow analysis with CFD, lift and thrust analysis, prototype construction, and flight tests. We came up with a final prototype in two versions, radio controlled and autonomous. Our prototype showed to be functional in the experimental tests, which include several flights with severe wind conditions in which the aircraft control showed to be smooth. To this end, we have a UAV with fully autonomous flight capability embarked, capable of acquiring images, and with the main characteristic that we named BROSH (Basic Risk Operation Self Handled) offering low risk to people and facilities.

Our aircraft has low cost for production and maintenance in comparison to other aircraft in operation. One of the main feature is its small size that represents another advantage. Our prototype can be constructed with less than US$ 500 per unit. It requires just one operator from launching and controlling it, eventually leaving it in autonomous mode close to landing, which is recommended to happen on a soft grass field. We notice that a micro UAV with the features described here is interesting in several application areas, such as monitoring in agriculture, monitoring of crowds (people or animals), border monitoring, surveillance and mapping, and searching of victims in disaster, between others.

In the near future, we will start applications with autonomous flight tests with image acquisition. Next, another commercial camera kit will be installed for transmission for integrated image acquisition with flight control. Notice that this allows to eventually change the programmed path on the flight, mainly in the case of beyond visual line of sight flight (BVLOS). Another very first work is with respect to the verification of its flight endurance (flight distance and time). Based on our calculations, we estimate that the current version is able to fly for at least 30 min, reaching a distance of little more than 20 km. We also plan to study and use visual model predictive control ^69^, which bases on visual information provided by the current camera for the control, and also testing our stochastic nonlinear model predictive control (SNMPC) algorithm for active target tracking ^69,70^. As a final idea of future work, we will develop an elastic launching platform for it, thus augmenting the pilot safety during the launching. Although a single operator can do that, a second operator launching it has proven to be safer.

## Data Availability

Data and design materials produced from or for this study can be requested to the authors by e-mail, including schematics of the original design that was submitted as a patent^45^” (see corresponding author e-mail).
